# Insight into the dimer dissociation process of the *Chromobacterium violaceum* (*S*)-selective amine transaminase

**DOI:** 10.1038/s41598-019-53177-3

**Published:** 2019-11-18

**Authors:** Federica Ruggieri, Jonatan C. Campillo-Brocal, Shan Chen, Maria S. Humble, Björn Walse, Derek T. Logan, Per Berglund

**Affiliations:** 1 Department of Industrial Biotechnology, KTH Royal Institute of Technology, AlbaNova University Center, SE-106 91 Stockholm, Sweden; 2 SARomics Biostructures AB, Medicon Village, SE-223 81 Lund, Sweden; 3 Pharem Biotech AB, Biovation Park, SE-151 36 Södertälje, Sweden

**Keywords:** Biocatalysis, Transferases, Enzyme mechanisms

## Abstract

One of the main factors hampering the implementation in industry of transaminase-based processes for the synthesis of enantiopure amines is their often low storage and operational stability. Our still limited understanding of the inactivation processes undermining the stability of wild-type transaminases represents an obstacle to improving their stability through enzyme engineering. In this paper we present a model describing the inactivation process of the well-characterized (*S*)-selective amine transaminase from *Chromobacterium violaceum*. The cornerstone of the model, supported by structural, computational, mutagenesis and biophysical data, is the central role of the catalytic lysine as a conformational switch. Upon breakage of the lysine-PLP Schiff base, the strain associated with the catalytically active lysine conformation is dissipated in a slow relaxation process capable of triggering the known structural rearrangements occurring in the holo-to-apo transition and ultimately promoting dimer dissociation. Due to the occurrence in the literature of similar PLP-dependent inactivation models valid for other non-transaminase enzymes belonging to the same fold-class, the role of the catalytic lysine as conformational switch might extend beyond the transaminase enzyme group and offer new insight to drive future non-trivial engineering strategies.

## Introduction

Amine transaminases (ATAs, EC 2.6.1.x) are enzymes with great potential in biocatalysis for the enantioselective synthesis of chiral primary amines from pro-chiral ketones^1,2^. Despite the high enantioselectivity and broad natural substrate scope of this enzyme class^3^, the constantly expanding toolbox of available (*R*)- and (*S*)-selective transaminases^3,4^ still has limited application in industrial processes^5^. One of the main factors limiting the implementation of transaminases on an industrial scale for the synthesis of chiral amines is their poor storage and operational stability^3,6^. While other properties such as substrate scope, enantioselectivity and inhibition have been successfully altered through enzyme engineering in a number of instances^3^, the improvement of the stability profile of transaminases has only gained momentum in the last couple of years. Although methods as diverse as semi-rational design^7^, structure-based re-design^8–11^, consensus mutagenesis^11,12^ and incorporation of unnatural amino acids^13^ have been successfully applied to improve the thermostability of some transaminases, we still do not have a clear understanding of the inactivation mechanism undermining the operational and storage stability of this group of enzymes. From an engineering perspective, a deeper insight into the molecular basis of transaminase inactivation could ease the identification of new mutagenesis targets for the improvement of transaminase stability.

**Figure 1:**
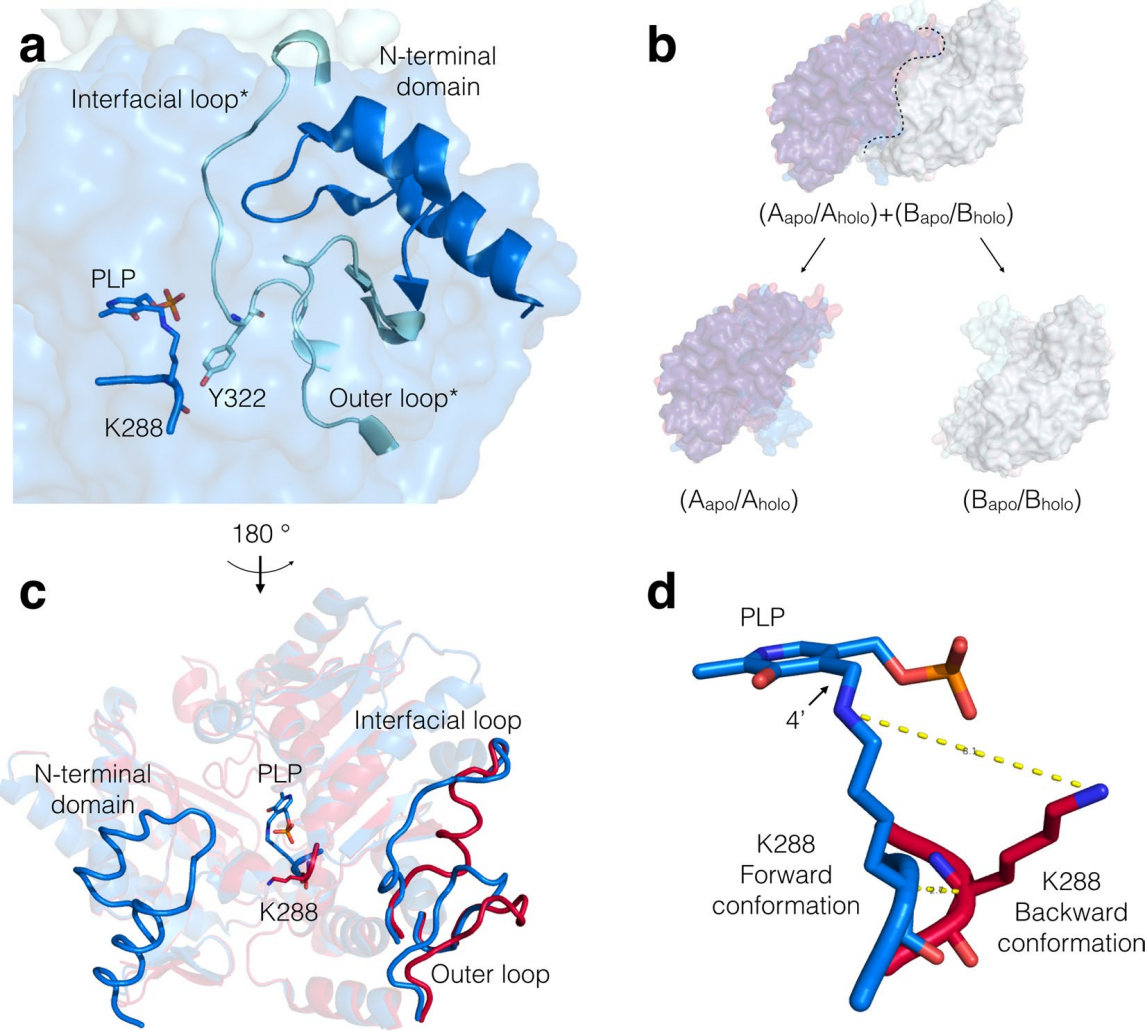
Conformational differences between the apo- and the holo-forms of the *Cv*-ATA, represented in pink and blue, respectively. The four variability regions are organized as a 3D interlock at the dimer interface of the holo-*Cv*-ATA (panel a). In each interlock the interfacial loop and the outer loop belonging to one monomer (shown in a lighter blue and indicated with *) are sandwiched between the PLP-K288 moiety and the N-terminal domain of the neighboring monomer (shown in a darker shade of blue). The superposition of the apo- and holo-*Cv*-ATA 3D structures represented as full surfaces (panel b) show that, upon PLP loss and structural rearrangement, the reorganization of the four variability regions results in significant alterations of the dimer interface. In each of the two monomers the “holo- to apo-” conformational changes consist of i) the disordering of the N-terminal domain; ii) the refolding of the interfacial loop away from the active site; iii) the rigid-body swing of the outer loop towards the bulk solvent and iv) the wide swing of the K288 side chain from a “forward” to a “backward” conformation, which also requires a deformation of the protein backbone (panels c and d).

Recently published data support the hypothesis of a key role for the cofactor pyridoxal-5′-phosphate (PLP) in the stability of both dimeric and tetrameric (*S*)-selective transaminases^6,14,15^, including the well-characterized homodimeric *Chromobacterium violaceum* (*S*)-ATA (*Cv*-ATA)^6,15^. In the last 25 years, a number of independent studies have investigated the stability and the folding dynamics of different fold type I PLP-dependent enzymes sharing the same overall folding and multimeric assembly as *Cv*-ATA^14–19^. These studies have revealed that, although the monomers of these enzymes fold independently of PLP^17–19^, both a structural rearrangement and the binding of the cofactor are necessary to produce an active holo-homodimer^19^. The further stabilizing role of PLP on the dimeric assembly, observed for different members of this fold class^15,19^, has been attributed to the network of inter-subunit polar interactions that PLP mediates across the dimer interface at the level of the phosphate group binding cups (PGBCs). In the *Cv*-ATA, the stabilizing role of PLP and, to a much lesser extent, inorganic phosphate, has also been attributed to the extensive H-bonding network established between the phosphate group of the cofactor and the residues of both subunits lining the PGBC in each of the two active sites^15,20^ (Supplementary, Figure S1). Structural data (Supplementary, Table S1 and Figure S2) have also revealed that upon PLP loss (i.e. in the transition from holo-*Cv*-ATA to apo-*Cv*-ATA) four “variability regions” organized in an interlock at the dimer interface (Fig. 1, panels a and b) undergo significant structural rearrangements^20^. These conformational changes consist of: i) the disordering in solution of the “N-terminal domain” (res. 5–35); ii) the reorientation of the catalytic K288 side chain (covalently bound *via* a Schiff base to PLP in the holo-enzyme) towards the back of the active site; iii) the outward rigid-body swing of the “outer loop” (res. 81–96) and iv) the recoiling and refolding of the “interfacial loop” (res. 311–326) away from the active site cavity (Fig. 1, panels c and d).

To the best of our knowledge, the functional significance of this extensive PLP-dependent conformational reorganization in the *Cv*-ATA has not been elucidated previously. In the present paper we propose a model explaining the role of PLP in dimer stability. Computational and experimental studies show that the stabilization role of PLP is exerted primarily at the level of the catalytic lysine K288 and secondarily at the level of the PGBC. This model unifies the available structural information and stability data and provides a new insight into the PLP-dependent dimer stability of *Cv*-ATA.

## Results and Discussion

### The PMP·*Cv*-ATA crystal structure

The transamination cycle catalyzed by *Cv*-ATA follows the general ping-pong bi-bi reaction mechanism accepted for transaminases^21^, relying on the formation of a reactive Schiff base between the side chain of the catalytic lysine (K288) and the 4′ group of the cofactor PLP (Fig. 1, panel d). The further participation of the K288 terminal amino group in catalysis as proton acceptor/donor (Supplementary, Scheme S1) requires its physical proximity to the 4′ group of the PLP ring. This proximity condition is not met in the apo-*Cv*-ATA conformation (PDB IDs 4A6R and 4BA4)^20,22^, where the side chain of the catalytic lysine is oriented towards the back of the active site, away from the 4′ group of the cofactor PLP. This K288 conformation will be here defined as the “K288 backward conformation” as opposed to the “K288 forward conformation” typical of the holo-*Cv*-ATA structures (PDB IDs 4A6T and 4AH3)^20^ (Fig. 1, panel d). The different K288 conformations and consideration of the proximity condition indispensable for catalysis led us to hypothesize that the apo-*Cv*-ATA 3D structures represent an inactivated state of the enzyme whose catalytic activity cannot be restored by simple cofactor replenishment unless conformational changes in the enzyme structure also occur.

In order to establish whether any of the variability regions undergo conformational changes during the catalytic cycle (i.e. upon transient disruption of the K288-PLP Schiff base), the crystal structure of the *Cv*-ATA in complex with the reaction intermediate pyridoxamine-5′-phosphate (PMP) (PMP·*Cv*-ATA) was solved at 1.67 Å resolution (PDB ID: 6S4G). The cryo-protection of single holo-*Cv*-ATA protein crystals in the presence of the amino donor 1-(*S*)-phenylethylamine (20 mM) led to the conversion of PLP to PMP *in cristallo* without producing any visible crystal cracking (Supplementary, Table S2).

The final refined structure model (Supplementary, Table S2) contains two homodimers per asymmetric unit, reciprocally oriented according to the same weak translational pseudo-symmetry described for the holo-form of the enzyme and each showing the same overall backbone folding^20^. The clear discontinuity in the electron density between the cofactor ring and the K288 side chain (Supplementary, Figure S3) justified the modeling of the cofactor moiety as PMP in all chains with 100% occupancy. In agreement with what has been observed for other PLP-dependent enzymes^23^, the conversion of PLP to PMP is associated with a small combined tilt and twist reorientation of the cofactor ring hinged on its pyridine nitrogen (Supplementary, Figure S4). The side chain of K288 was modeled with 100% occupancy in the forward conformation with only the three most terminal side chain atoms tilted away from the amino group of PMP (Supplementary, Figure S5).

The PMP·*Cv*-ATA structure shows that, upon breakage of the K288-PLP Schiff base linkage, neither K288 nor the other variability regions rearrange to their apo-conformation on the seconds time scale of cryoprotection. Since the actual reaction turnover is expected to be orders of magnitude faster, it can be concluded that these structural rearrangements do not occur during the catalytic cycle and must be the outcome of a different process.

### The PLP-dependent *Cv*-ATA inactivation model

Differently to the hypothesis that the phosphate group of PLP is the main contributor to dimer stability^15^, we propose for the first time that, at least in the case of the *Cv*-ATA, the stabilizing role of PLP is mainly exerted at the level of the catalytic lysine and secondarily at the level of the PGBCs. Indeed, from inspection of the Ramachandran plots of the available *Cv*-ATA structures, we could conclude that the forward K288 conformation, contrary to the backward conformation, requires residues A287 and K288 to fall in the least energetically favourable regions of the plot (Supplementary, Figure S6). The resulting backbone tension is compensated by the K288-PLP Schiff base, which strains the K288 in the forward conformation required for catalysis. Upon PLP loss, the Schiff base restraint is lifted and the rearrangement of K288 to the more energetically favorable backward conformation is enabled. We further propose that this K288 rearrangement is the conformational switch triggering the structural changes in the neighboring variability regions across the dimer interface in a cascade inactivation process that culminates in dimer dissociation. Indeed, the steric clash between the rearranging K288 and the bulky side chain of Y322* (* denotes elements belonging to the neighboring monomer) (Supplementary, Figure S7) would trigger the recoiling of the interfacial loop* (on which Y322* is located) away from the active site. The recoiling of this loop would in turn displace the outer loop* and the N-terminal domain. The loss of order of the latter element, which is likely to contribute to dimer stability by clamping the interfacial* and outer loop* on the active site cavity (Fig. 1, panel a), is in agreement with the lack of electron density for this region in all known apo-*Cv*-ATA structures. The displacement of the N-terminal domains, in combination with the opening of two solvent-accessible clefts in the space opened by the two recoiled interfacial loops (Supplementary, Figure S8), would contribute to destabilize the dimer assembly.

The proposed model also accounts for the previously described stabilizing role of the extensive PGBC H-bonding network, which helps prevent PLP loss by anchoring it inside the active site and provides the polar interactions needed to keep the interfacial loop stretched inside the active site, hence hindering the K288 rearrangement. The model accounts for all of the conformational changes observed in the transition from holo- to apo- enzyme and for the experimentally observed role of PLP and inorganic phosphate in the stabilization of the monomer-monomer association.

### Computational validation of the *Cv*-ATA inactivation model

**Figure 2:**
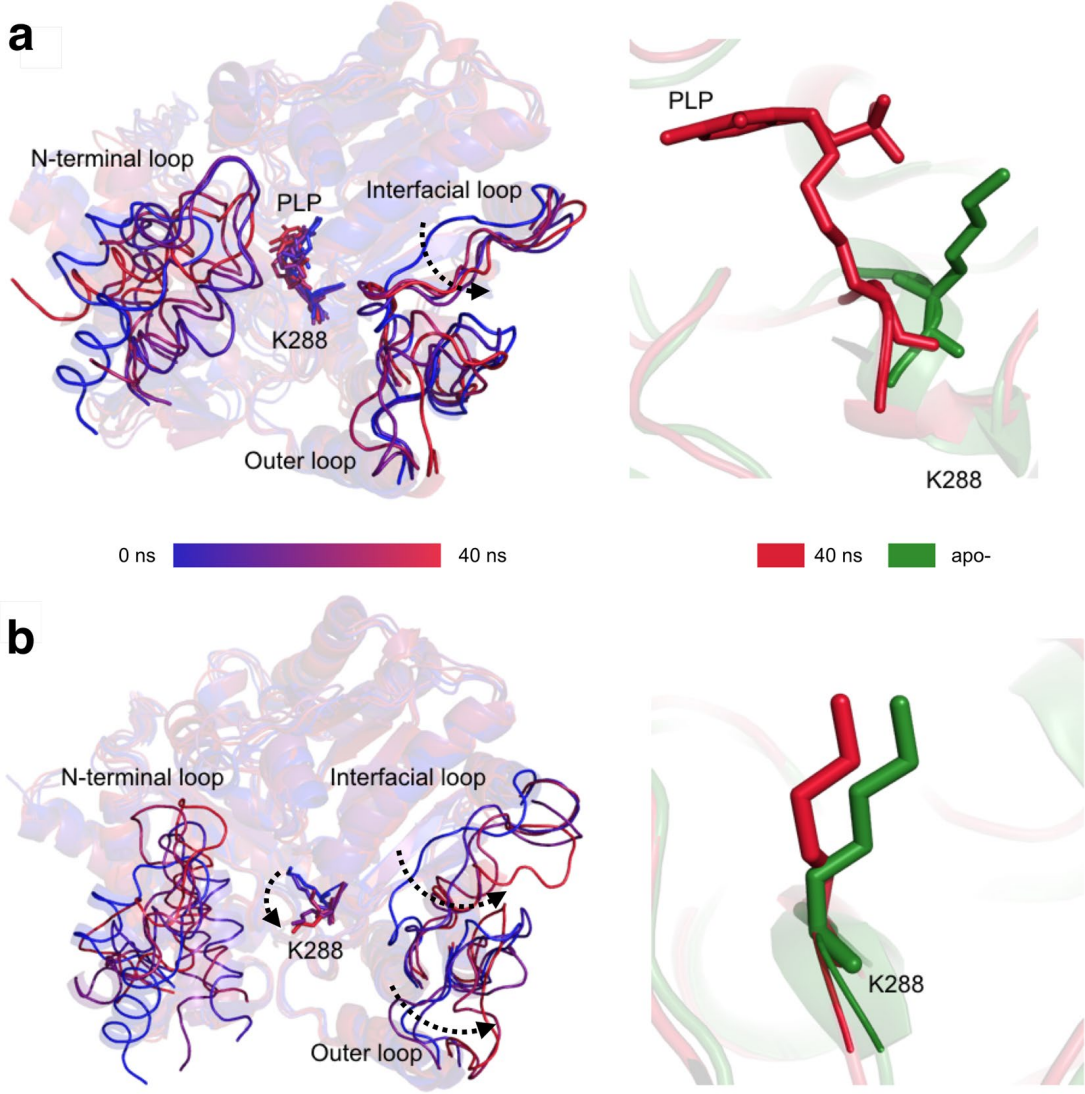
Results of the first computational validation step for the proposed *Cv*-ATA inactivation model performed on the isolated holo-monomer (PDB ID 4A6T, chain A) in the presence (panel a) or absence (panel b) of the PLP moiety. The superposed snapshots of the MD simulations (0 ns, 10 ns, 20 ns, 30 ns and 40 ns) are shown on the left-hand side of each panel in a progressive color palette going from blue to red. Significant directional rearrangements are indicated with discontinuous black arrows. The comparison between the backward K288 conformation of the apo-*Cv*-ATA (PDB ID 4A6R, chain A, in green) and the K288 conformation at the end of each MD simulation (40 ns, in red) is shown on the right-hand side of each panel.

The proposed *Cv*-ATA inactivation model was computationally tested in three steps by molecular dynamics (MD). In the first validation step, the effect of PLP loss on the conformation of the catalytic lysine K288 was isolated from its effect on the conformation of the interfacial loop* by performing MD on the isolated holo-*Cv*-ATA monomer considered with or without the PLP atoms. Our results show that, while the K288 rearranges completely to the backward conformation upon removal of PLP, the K288-PLP Schiff base effectively prevents this rearrangement under the same simulation conditions (Fig. 2). During the course of both simulations, the other variability regions belonging to the same monomer (N-terminal domain, interfacial loop and outer loop) are found to be flexible, probably due to the absence of monomer-monomer contacts and/or PGBC interactions (Fig. 2).

**Figure 3:**
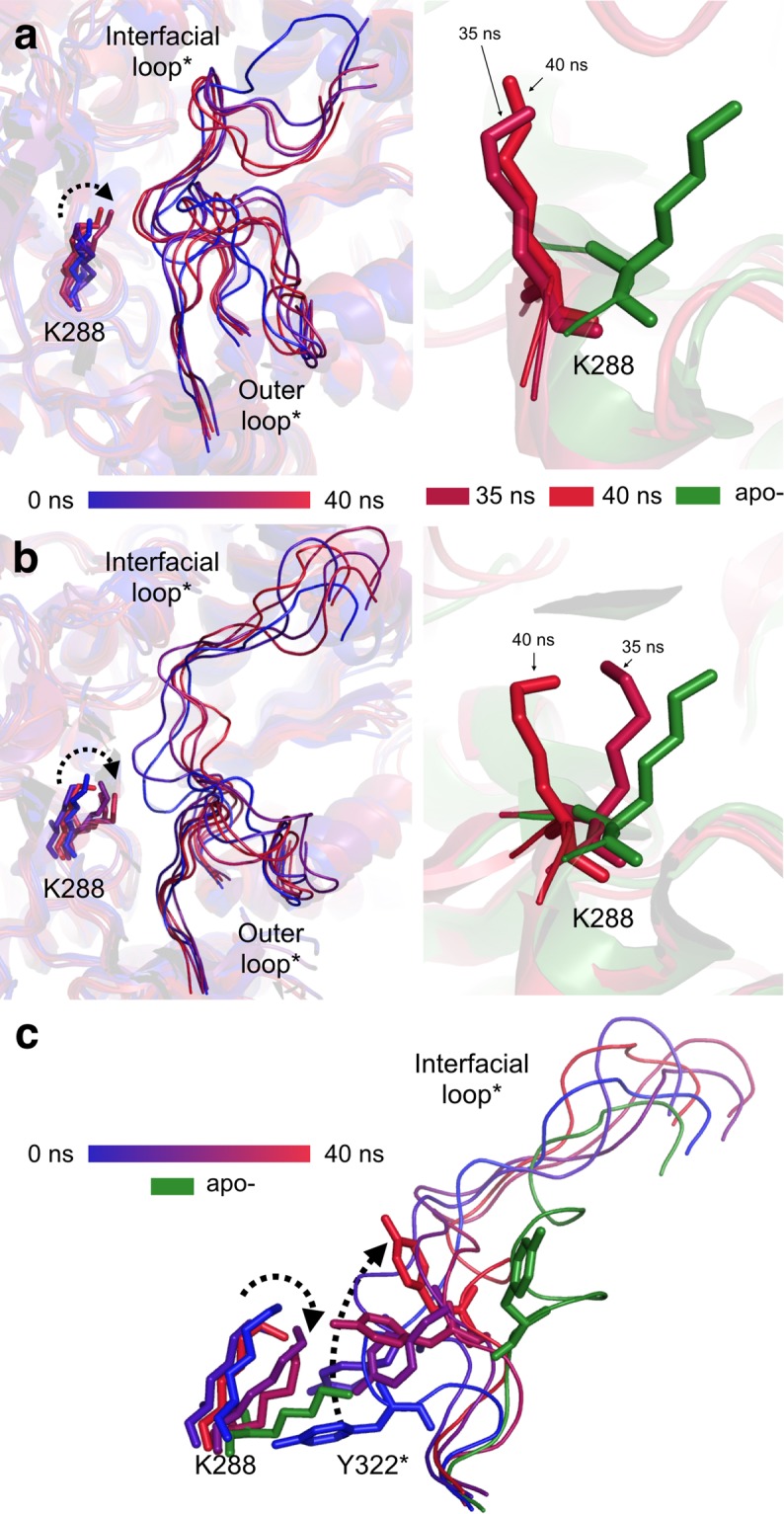
Results of the second computational validation step for the proposed Cv-ATA inactivation model performed on the holo- dimer (PDB ID 4A6T, chains AB). The left-hand side of panels a and b shows the superposition of the MD simulation snapshots (0 ns, 10 ns, 20 ns, 30 ns and 40 ns is in a progressive color palette going from blue to red) for active site A and B, respectively. The right-hand side of panels a and b shows the comparison between the K288 conformations in the last two simulation snapshots (35 ns and 40 ns, labelled and represented in different shades of red) and the backward K288 conformation characteristic of the apo-*Cv*-ATA. The dynamic interplay of the K288 and the interfacial loop* in active site B is shown in panel c. The side chain orientations of K288 and Y322* (shown as sticks) are shown in the same progressive color palette used for panels a and b as they rearrange during the course of the simulation. Significant directional rearrangements are indicated with discontinuous black arrows.

In the second validation step, the dynamic interplay between K288- and the interfacial loop* conformations was evaluated by simulating the effect of PLP loss on the *Cv*-ATA holo-dimer (PDB ID 4A6T, dimer AB). In this step, as well as in the third validation step, the computational load of the MD calculation was reduced by deleting the atomic coordinates corresponding to the two N-terminal domains (res. 5–35), which are disordered in the apo-*Cv*-ATA and are not directly involved in the K288-interfacial loop* interplay. Over the time of the simulation (40 ns), the two active sites (herein defined A or B depending on the chain contributing the K288 residue) showed different MD progressions, possibly related  to their different starting crystallographic B-factors (Supplementary, Table S3). While neither K288 or the interfacial loop* belonging to the active site A rearranged significantly during the course of the simulation (Fig. 3, panel a), both the K288 and the interfacial loop* belonging to the active site B (characterized by a higher crystallographic B-factor) rearranged almost completely to the backward and recoiled conformations, respectively (Fig. 3, panels b and c).

**Figure 4:**
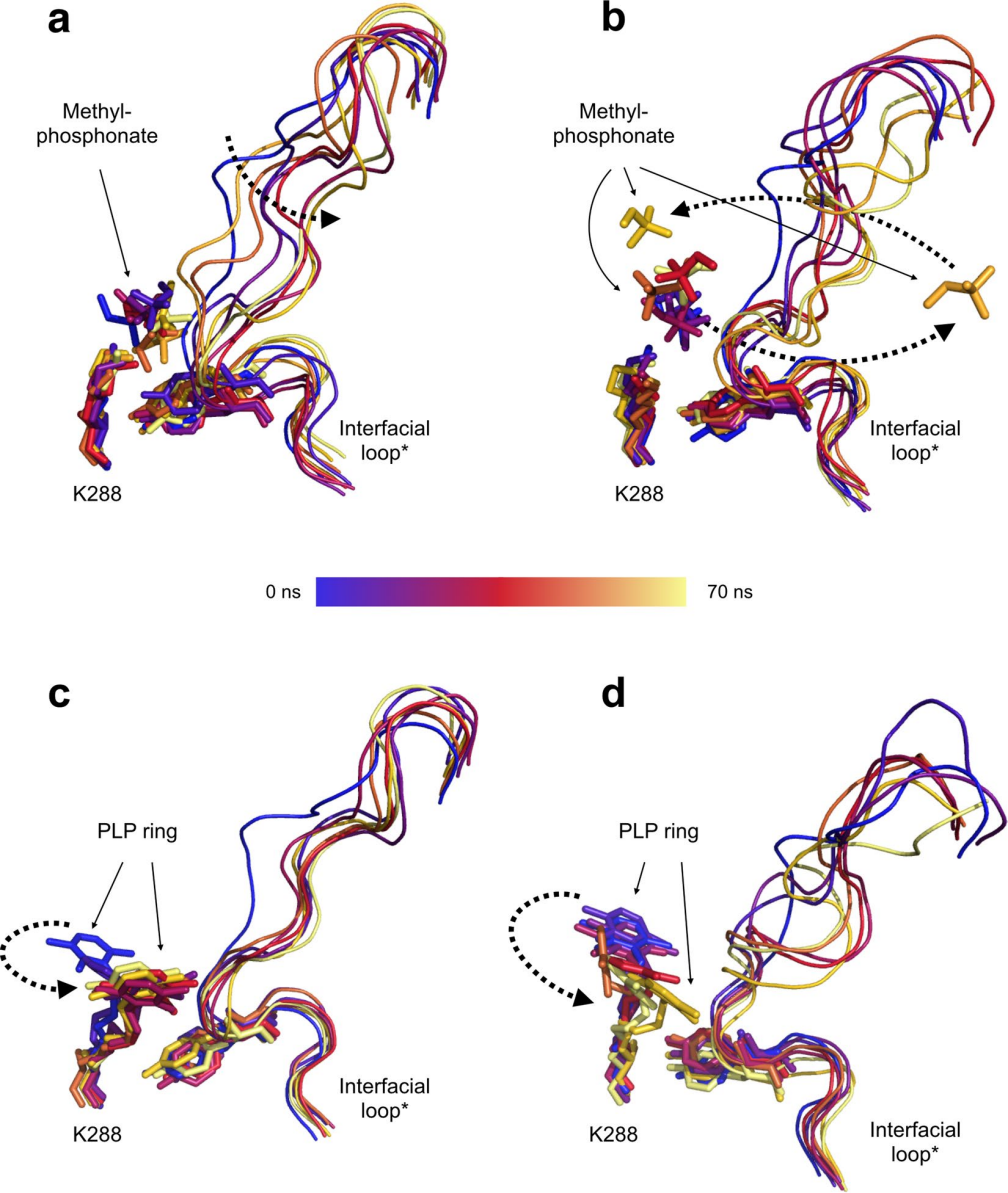
Results of the third computational validation step for the proposed *Cv*-ATA inactivation model performed on the holo- dimer (PDB ID 4A6T, chains AB). Panels a and b show the MD snapshots (0 ns, 10 ns, 20 ns, 30 ns, 40 ns, 50 ns, 55 ns, 60 ns, 70 ns, progressive color palette blue-red-yellow) for the case of the coordinated methyl phosphonate in monomers A (panel a) and B (panel b). The coordinated phosphonate in the PGBC (panel a) rigidifies the conformation of the interfacial loop* inside the active site and hinders the rearrangement of the K288. The diffusion of the phosphonate away from the PGBC (panel b) makes the movement of the free K288 possible. Panels c and d show the MD snapshots (0 ns, 10 ns, 20 ns, 30 ns, 40 ns, 50 ns, 55 ns, 60 ns, 70 ns, progressive color palette blue-red-yellow) for the case of the phosphate-depleted PLP bound to the K288 *via* the Schiff base. The lack of coordinated phosphate, after inducing an initial reorganization of the interfacial loop*, is not observed to cause mayor conformational rearrangements in this region. During the course of the same simulation time, the K288, bound *via* the Schiff base to the PLP ring, rearranges towards the backward conformation. Significant directional rearrangements are indicated with discontinuous black arrows.

In the third and last validation step, the roles of the two cofactor substructures (i.e. the 5′-phosphate group and the 4′ group involved in Schiff base formation) in the active-site conformational changes were isolated and investigated independently in MD simulations (70 ns) performed on the *Cv*-ATA homodimer (PDB ID 4A6T, dimer AB). In the first case, the deletion of the cofactor pyridine ring generated the methylphosphonate ligand, which was used as a mimic for the 5′-phosphate group of PLP. The coordination of the methylphosphonate in the PGBC was observed to hinder the rearrangement of K288 to the backward conformation (Fig. 4, panels a and b). This is likely to depend both on the effect of coordination of the methylphosphonate on the rearrangement trajectory of K288, which interferes with its side chain reorientation, and on the network of H-bond interactions spanning the PGBC that “pull” the interfacial loop* inside the active site, rigidifying it and impeding its K288-mediated displacement. The loose coordination of the methylphosphonate ligand (Fig. 4, panels a and b), which can diffuse out of the active site cavity (Fig. 4, panel b), is therefore expected to affect the *Cv*-ATA stability by slowing down the rate of the K288 rearrangement. In the second case, the deletion of the 5′-phosphate atoms from PLP generated the PLP-desphosphate moiety bound *via* the Schiff base to the side chain of K288. Our MD results led us to conclude that the Schiff base alone is not sufficient to prevent the rearrangement of K288 to the backward conformation (Fig. 4, panels c and d). Indeed, the K288 backbone strain is predicted to be sufficient to trigger the rearrangement of the K288 bound *via* the Schiff base to the PLP ring, although this rearrangement is expected to be slower than in the case of the free K288. In one of the chains (chain B, Fig. 4, panel d, 60 ns), and in agreement with the results of the other simulations, the displacement of the K288-Schiff base was observed to trigger a backbone deformation in the Y322* ± 1 region, occurring at the same time as a sudden recoiling in the N-terminal portion of the interfacial loop*.

Our MD studies support the proposed inactivation model by showing that (i) the PLP removal in the holo-*Cv*-ATA monomer triggers the K288 conformational rearrangement to the backward conformation; (ii) in the holo-*Cv*-ATA dimer, this rearrangement leads to a steric clash between the rearranging K288 and the Y322* side chains, which triggers the recoiling of the interfacial loop*; (iii) the K288 backbone tension cannot be compensated by the Schiff base linkage alone and the broad interaction network in which PLP phosphate group participates to  further rigidify the Schiff base linkage by anchoring the cofactor in the active site; (iv) the presence of a (loosely) coordinated phosphonate in the PGBC creates sufficient steric hindrance and interfacial loop* rigidity to prevent the K288 rearrangement, thus contributing to the *Cv*-ATA stability, as reported in previous studies^15^.

### Experimental investigation of the role of the Schiff base in the *Cv*-ATA stability

The roles of the K288-PLP Schiff base linkage and of the bulky Y322 side chain on the thermodynamic stability of the *Cv*-ATA were experimentally investigated by melting point measurements, mutagenesis studies and combinations thereof. For this purpose, two enzyme variants with expected altered stability profiles were designed: one (K288A) intrinsically unfit to bind PLP through formation of the Schiff base and the other (Y322A) minimizing the clash between the rearranging K288 and the interfacial loop*. The melting temperatures (*T*_m_) of the resulting Schiff base-deficient K288A mutant and of the less sterically hindered Y322A variant were thereafter measured by DSF^24^ and compared to the corresponding values in the wild-type (WT) *Cv*-ATA.

**Figure 5:**
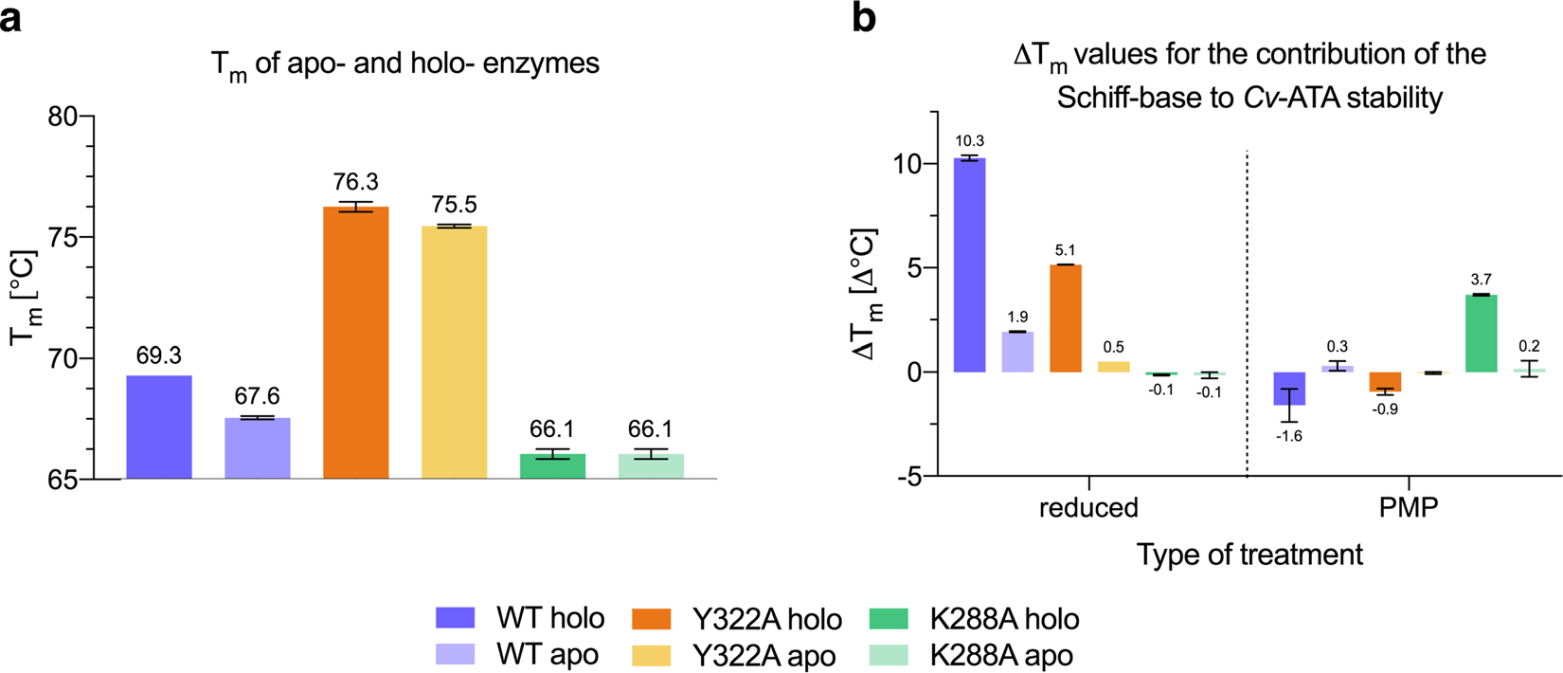
Results of the melting temperature (*T*_m_) measurements for the untreated (panel a) and treated (panel b) apo- and holo- *Cv*-ATA wild-type (WT), Y322A and K288A. The values for the untreated samples are shown as absolute values of *T*_m_ expressed in °C. The values for the treated samples (either reduced with the reducing agent NaCNBH_3_ or incubated in the presence of amino donor L-alanine to form non-covalently coordinated PMP) are reported as *T*_m_ shifts (Δ*T*_m_) expressed in Δ°C.

The measured *T*_*m*_ values for the holo-*Cv*-ATA wild-type (WT), Y322A and K288A (holo- *T*_m_ values) are 69.3 °C, 76.2 °C and 66.1 °C, respectively. These values show that, while the mutation of the catalytic lysine K288 into an alanine has a mild destabilizing effect (Δ*T*_m(K288Aholo-WTholo)_ = −3.2 °C; Δ*T*_m(K288Aapo-WTapo)_ = −1.5 °C), the mutation of Y322 into an alanine correlates with increased protein stability (Δ*T*_m(Y322Aholo-WTholo)_ =  + 6.9 °C; Δ*T*_m(Y322Aapo-WTapo)_ =  + 7.9 °C) (Fig. 5, panel a). This latter observation can be explained by the increased space available in the Y322A to accommodate the reorientation of the K288 side chain released upon uncatalyzed Schiff base hydrolysis^25^, thus preventing the recoiling of the interfacial loop* and the consequent weakening of the monomer-monomer interface. This result shows that the alanine mutation in position 322 outbalances the loss of the polar interactions mediated by the Y322 side chain hydroxyl group and supports the hypothesis that the monomer-monomer clash caused by the K288 reorientation is the main factor affecting *Cv*-ATA stability.

The *Cv*-ATA WT and Y322A variants, both expected to form a Schiff base with PLP, displayed an increased *T*_m_ compared to their apo-forms, Δ*T*_m(WTholo-WTapo)_ = + 1.8 °C and Δ*T*_m(Y322Aholo-Y322Aapo)_ = + 0.8 °C, which supports the hypothesis of a stabilizing effect of the K288-PLP Schiff base linkage (Fig. 5, panel a). The *T*_m_ of the K288A variant incubated with PLP (K288A_holo_) was not significantly different from the *T*_m_ of the non-incubated sample (K288A_apo_).

**Figure 6:**
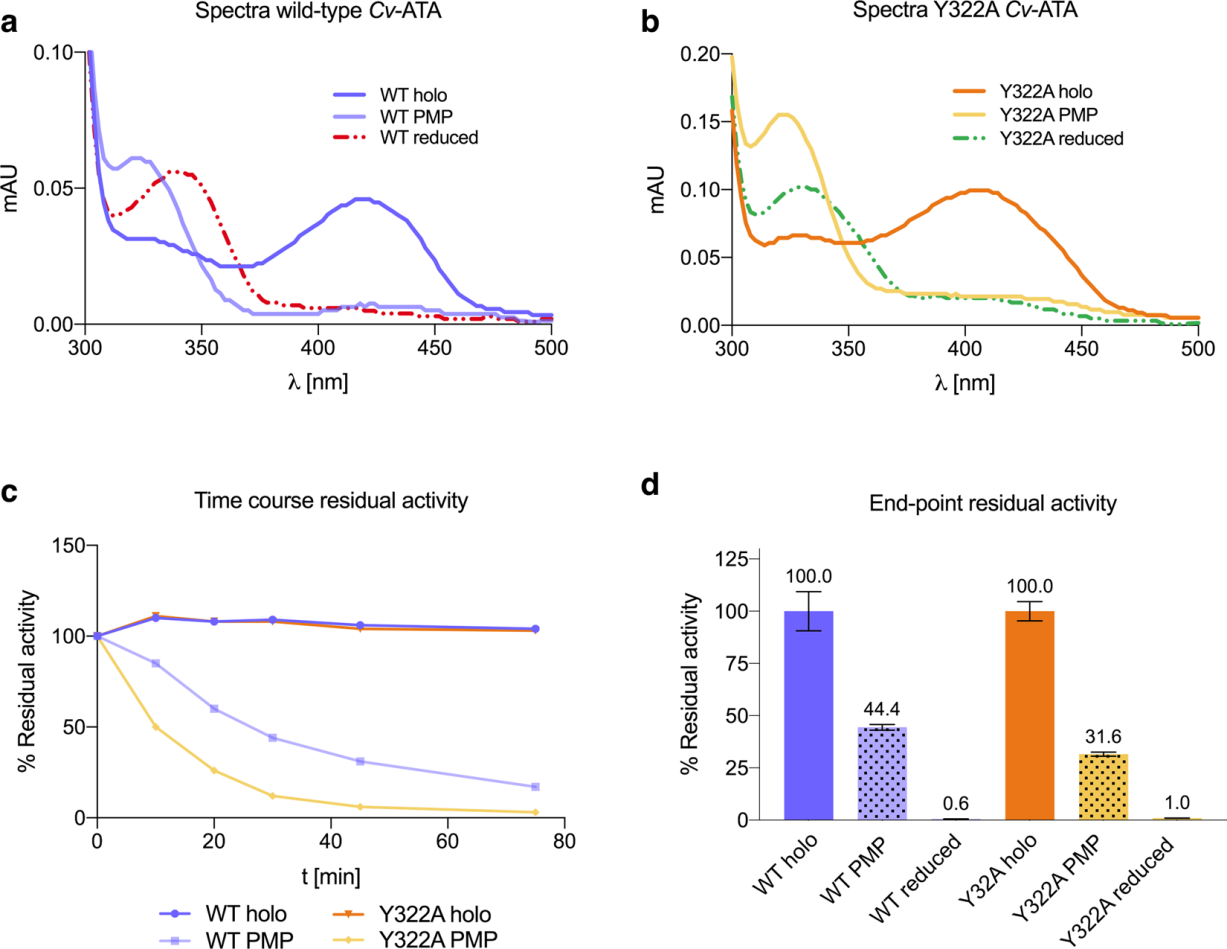
Results of the Schiff base treatments (reduction to non-hydrolyzable secondary amine and conversion to non-covalently bound PMP) on the spectroscopic and activity profile of the two catalytically active wild-type (WT) and Y322A *Cv*-ATA. For both enzymes, the modification of the Schiff base using either of the two methods resulted in the red-shift of the absorption maximum characteristic of the Schiff base (~420 nm) (panels a and b, referring to the *Cv*-ATA WT and Y322A, respectively). For both enzymes the conversion of the cofactor PLP to non-covalently bound PMP at room temperature in the absence of amino donors resulted in the rapid decrease in activity of both *Cv*-ATA enzyme variants, WT and Y322A (panel c). The incubation in the presence of either amino donor or reducing agent (4 h, on ice) show that for both enzymes the catalytic activity is irreversibly lost (panel d). While the reduction of the Schiff base completely obliterates the enzymatic activity, the conversion to PMP in the absence of an amino acceptor reduces the enzymatic activity to 44.4% and 31.6% for the *Cv*-ATA WT and Y322A, respectively.

The role of the K288-PLP Schiff base in *Cv*-ATA stability was further tested by chemical modification of the Schiff base to a non-hydrolyzable secondary amine using the selective reducing agent NaCNBH_3_ to form the “reduced” form of the enzyme^17,26^. The chemical modification, which causes the complete loss of enzymatic activity and a red shift in the UV absorption maximum characteristic of the Schiff base (Fig. 6, panels a and b), had no effect on the *T*_m_ of the K288A variant, either in the apo- or in the holo-form. On the contrary, the NaCNBH_3_-assisted Schiff base reduction resulted in a significant stabilization of the holo-*Cv*-ATA WT and Y322A, with Δ*T*_m(WTholo(reduced)-WTholo)_ = 10.3 °C and Δ*T*_m(Y322Aholo(reduced)-Y322Aholo)_ = 5.2 °C (Fig. 5, panel b). The stabilization observed for apo-*Cv*-ATA WT and Y322A upon treatment with NaCNBH_3_ (Δ*T*_m_ = 2 °C and 0.5 °C, respectively) can be explained by the presence of a variable, small fraction of holo-enzyme in the purified apo-protein preparations^15^.

Lastly, the effect of amino-donors on *Cv*-ATA stability was also investigated in a set of DSF experiments. Recently Börner *et al*.^14^ reported that *Cv*-ATA incubation in the presence of an amine substrate results in enzyme de-stabilization and explained this observation with the possible diffusion of the non-covalently bound PMP away from the active site. This is in agreement with our model, as the conversion of PLP to PMP releases the K288 from the Schiff base constraint and enables the K288 reorientation to the backward conformation. Indeed, our results show that prolonged incubation in the presence of L-alanine (reactive towards the K288-PLP Schiff base to form the enzyme·PMP complex) resulted in decreased stability for both the WT and the Y322A *Cv*-ATA, with Δ*T*_m(WTholo-WT·PMP)_ = −1.6 °C and Δ*T*_m(Y322Aholo-Y322A·PMP)_ = −0.9 °C (Fig. 5, panel b). The lifting of restraints on K288 upon formation of non-covalently associated and diffusible PMP was also found to correlate with irreversible loss of activity (Fig. 6, panels c and d). This result confirms that prolonged Schiff base disruption leads to irreversible enzyme inactivation in *Cv*-ATA. Similarly to what was shown in the MD simulations in the presence of methyl phosphonate, the diffusion of the PMP molecule away from the active site enables the structural rearrangements leading to the apo- conformation of the enzyme and culminating in dimer dissociation. The stabilization of holo-K288A observed upon incubation with L-alanine (Δ*T*_m_ = 3.7 °C) (Fig. 5, panel b) can be explained by the non-covalent coordination in the enzyme active site of a spontaneously formed L-alanine-PLP external aldimine species (Supplementary, Scheme S1), locked in place *via* the PGBC interactions. This result prompted us to investigate further the effect on stability of non-covalently bound species.

### Experimental investigation of the role of non-covalent coordination on *Cv*-ATA stability

The principle of ligand-induced protein stabilization is now widely accepted and routinely used, among other applications, in the screening of drug compounds^27^.

Recently, Chen *et al*.^15^ have applied this principle to demonstrate the stabilizing effect of PLP and inorganic phosphate on the *Cv*-ATA. The results we collected for the MD simulation performed in the presence of methyl phosphonate (Fig. 4) support these data and suggest that the coordination of a phosphate ion could promote the stability of the dimer. Indeed, as discussed previously, by hindering the K288 rearrangement to the backward conformation and by rigidifying the interfacial loop* in its extended (uncoiled) conformation, the phosphate ion, just like the methylphosphonate, would prevent the structural rearrangements occurring at the dimer interface responsible for the weakening of the monomer-monomer interactions (Supplementary, Figure S8).

**Figure 7:**
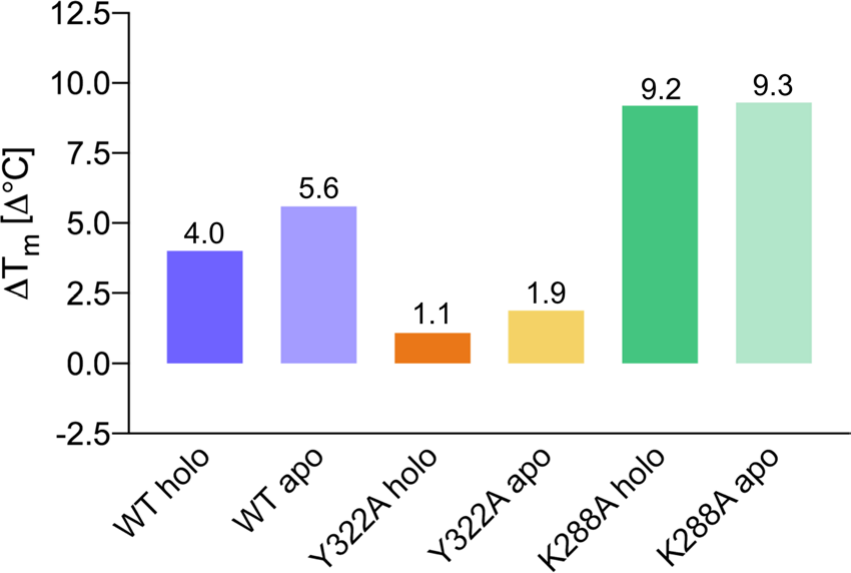
*T*_m_ shifts measured by DSF on the holo- and apo- *Cv*-ATA WT, K288A and Y322A incubated in the presence of equimolar amounts of excess amino donor L-alanine and PLP.

Our *T*_m_ measurements (Fig. 5, panel b) also suggested that non-covalent, spontaneous external Schiff bases formed in solution when excess PLP and amine substrate are present can enhance the stability of the Schiff base-deficient K288A *Cv*-ATA variant, possibly thanks to non-covalent interactions established inside the PGBC and/or across the active site cavity. Indeed, the incubation of all three enzyme variants (*Cv*-ATA WT, Y322A and K288A) in the presence of equimolar amounts of L-alanine and PLP (20 mM) led to positive *T*_m_ shifts (Fig. 7). The largest stabilizing Δ*T*_m_ is measured for the apo-forms of the Schiff base-competent WT and Y322A variants where the bulky, phosphate-containing external aldimine species does not compete with covalently bound PLP for the active sites. The identical contribution to stability in the case of the apo- and holo- K288A reinforces the hypothesis that this type of spontaneously formed ligand can bind non-covalently to catalytically inactive enzymes and contribute to their stabilization.

We suspect that this is what happened in the case of the deposited crystal complex between *Cv*-ATA and the dead-end inhibitor gabaculine (Supplementary, Table S1 and Figure S2, PDB ID 4BA5, GABA·*Cv*-ATA)^22,28^. The extensive similarities between the GABA·*Cv*-ATA and the apo-*Cv*-ATA structures (i.e. the lack of density for the N-terminal domain, the K288 in the backward conformation and the interfacial loops modeled in different stages of recoiling (Supplementary, Figure S9)), indicate that the GABA·*Cv*-ATA structure could be the true complex of the apo-*Cv*-ATA enzyme binding the dead-end PLP-gabaculine adduct. If this were true, the GABA·*Cv*-ATA crystal complex would also demonstrate that the presence of a coordinated phosphate group, regardless of the number and type of interactions established by the rest of the ligand structure, is not sufficient to prevent the holo-to-apo structural rearrangements leading to enzyme inactivation (and ultimately dimer dissociation). Indeed, this is what can be observed in the case of the large, phosphate-containing external-aldimine-like PLP-gabaculine adduct. The extensive structural similarities between the GABA·*Cv*-ATA crystal complex and the apo-*Cv*-ATA crystal structure, jointly with the different geometrical and electronic properties of the gabaculine-PLP adduct with respect to those expected for the PLP-gabaculine transition-state (Supplementary, Figure S10), led us to question the protein-ligand interaction space determined based on the GABA·*Cv*-ATA crystal complex. In order to gather information about the true transition state stabilization in the catalytically active enzyme, *in silico* docking experiments were performed (Supplementary, Figure S11). The results showed that the binding of the expected PLP-transition state in the holo-*Cv*-ATA active site does not require any of the active site rearrangements observed in the GABA-*Cv*-ATA crystal complex.

### Structural analysis of the interfacial loop conformations in other PLP fold type I enzymes

Finally, the structural conservation of the *Cv*-TA interfacial loop was investigated using a subset of PLP-dependent fold type I enzymes retrieved by a PDB-wide structure search. The initial 2187 redundant hits retrieved by a structure search using the Dali server^29^ were manually curated to select a working set of 22 structures showing 59 to 12% structural identity to *Cv*-TA (Supplementary, Table S4). In the light of our findings regarding the possibility of structural reorganizations *in cristallo* upon Lys-PLP Schiff base disruption, PMP and inhibitor complexes were excluded from the working set. Engineered variants and ancient enzyme reconstructions were also excluded. For each selected hit, the PDB was manually searched to retrieve, when possible, both the apo- and the holo- crystal structure. The final working set contained the structures of 17 transferases (16 transaminases and 1 desulfurase) and 1 epimerase, giving a total of 8 apo- and 14 holo-structures presenting varying quaternary crystallographic assembly (monomers, dimers, tetramers and dimers of dimers) (Supplementary, Table S4). The working set included enzymes isolated from mesophiles, thermophiles and halophiles and covered different regions of the homology network elaborated by Percudani *et al*.^30^.

While in most instances only the crystal structure of one enzyme form has been deposited in the RCSB PDB, for the *Vibrio fluvialis* transaminase (*Vf-*TA), the *Lactobacillus buchneri* isoleucine 2-epimerase (*Lb-*IE) and the *Pseudomonas jersenii* transaminase (*Pj-*TA) both the apo- and the holo-structure are available (Supplementary, Table S4). The reliability of the structures in the important regions was verified by inspection of the electron density for the interfacial loop and the catalytic lysine residue, using maps downloaded from the PDB_REDO server^31^. In all cases, the deposited model was found to be a reliable reflection of the experimental maps.

The structural analysis revealed that also in *Vf-*TA and *Lb*-IE (containing an alanine and a threonine as structural analogues to the *Cv*-TA Y322, respectively) the interfacial loop rearranges (*Vf*-TA) or becomes disordered (*Lb*-IE) upon PLP loss (Supplementary, Figure S12). The mode of interfacial loop rearrangement appears to be enzyme-specific and most likely sequence-dependent, particularly with respect to, but not limited to, the structural analogue to the *Cv*-TA Y322. On the other hand, in the case of *Pj*-TA, where both this residue and the residue preceding the strained catalytic lysine are glycines, no interfacial loop reorganization is observed upon cofactor loss (Supplementary, Figure S12).

The observation that the interfacial loop rearrangement may be sequence-dependent led us to investigate the degree of amino acid variation among the *Cv*-TA Y322 structural analogues (Supplementary, Figure S13). The successful multiple sequence alignment of 17 of the 18 unique sequences belonging to the working set revealed that in 13 instances the structural analogue to the *Cv*-TA Y322 is a tyrosine, in two instances it is an alanine and in three instances if is either a threonine, a glycine or a phenylalanine (Supplementary, Table S4 and Figure S13). The only sequence producing non-significant alignments belonged to the *Hydrogenomonas thermophilia* desulfurase (*Ht*-DS), which also showed the lowest structural identity to *Cv*-TA (12%). A structure alignment between the holo-*Cv*-TA and the holo-*Ht*-DS (PDB ID: 5ZSP) revealed that, in this enzyme, the interfacial loop region has a different architecture to that previously described for *Cv*-TA (Supplementary, Figure S14). Hence, no clear structural analogue to the *Cv*-TA Y322 can be identified for this particular enzyme. In addition, no backbone strain seems to be associated with the conformation of the catalytic lysine in this enzyme.

The finding that one PLP-dependent fold-type I enzyme (*Ht*-DS) presents a different interfacial loop architecture prompted us to investigate the degree of structural conservation of the interfacial loop among the holo-structures in the working set (Supplementary, Table S4). An all-against-all structure alignment revealed a high structural conservation around the *Cv*-TA Y322 position (and higher structural divergence in N-terminal portion of the interfacial loops) in all of the considered holo-structures, with the only exception of the *Salmonella thyphimurium* transaminase (*St*-TA), where the interfacial loop appears to be disordered (Supplementary, Figure S15, panel a). The analysis of the Ramachandran plots for these holo-structures revealed that in all cases the catalytic lysine and its preceding residue fall in the least energetically favourable regions. A similar all-against-all comparison was finally performed considering all of the apo-structures included in the working set (Supplementary, Figure S15, panel b). In this case, very little structural conservation could be observed, as the interfacial loops are found to be either disordered (completely or in part) or folded away from the PGBC. The only three exceptions are represented by the *Bacillus anthracis* and *Silicibacter pomeroyi* transaminases (*Ba-*TA and *Sp*-TA) and by the previously mentioned *Pseudomonas jersenii* transaminase (*Pj-*TA). Similarly to what was shown in the MD simulation in the presence of methyl phosphonate, and in agreement with previous hypotheses^20,32^, the coordination of a sulfate ion in the PGBC of *Ba-*TA and *Sp*-TA might have hindered the rearrangement of the catalytic lysine in these apo-structures, while the presence of two glycine residues as both structural analogue to the interfacial loop tyrosine and residue preceding the catalytic lysine might reduce the backbone strain in *Pj-*TA. In the remaining cases, incomplete electron densities for the interfacial loops might indicate the coexistence in the crystals of different stages of holo-to-apo rearrangements. For these reasons, a Ramachandran plot analysis appears to be less informative in the case of the apo-structures considered in this study.

The findings of this structure analysis suggest that PLP-dependent interfacial loop rearrangements similar to those investigated in this study for *Cv*-TA might occur for other enzymes belonging to the same fold type, even non-transaminase, with possibly a few noteworthy exceptions.

## Conclusion

Despite their great potential for the synthesis of enantiopure chiral amines on an industrial scale, one of the main obstacles to the use of wild-type amine transaminases (EC 2.6.1.x) for biocatalytic applications is their often poor operational and storage stability. Our scarce understanding of the inactivation mechanism of these enzymes limits the use of rational design approaches targeting the enhancement of their stability.

**Figure 8:**
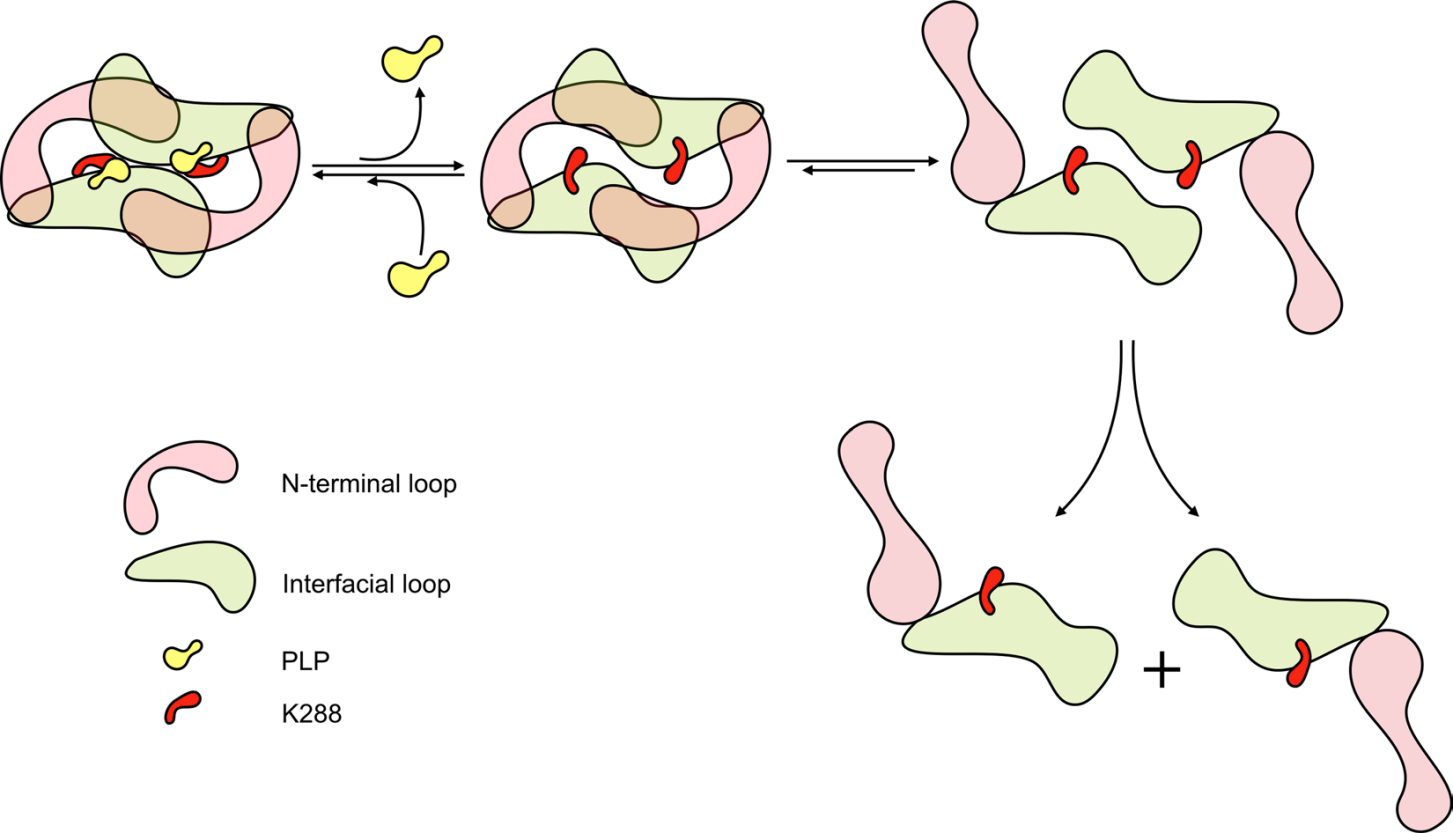
Schematic representation of the proposed *Cv*-ATA inactivation mechanism, i.e. transition from holo- to apo-form. The first event in the inactivation process is the loss of cofactor PLP, accompanied by the disruption of both the K288-PLP covalent Schiff base and the hydrogen bonding network in the PGBC. The relaxation of the K288 to the backward conformation, is enabled by the loss of rigidity of the interfacial loop*. The recoiling of the interfacial loop triggers the displacement of the outer loop (not represented) and the N-terminal domain thereby causing the opening of two solvent-accessible clefts. The decreased monomer-monomer association energy enables dimer dissociation in solution.

In the present paper we have described and validated a possible inactivation model for the well-characterized homodimeric (*S*)-selective amine transaminase from *Chromobacterium violaceum* (Fig. 8). The starting point for the proposed *Cv*-inactivation model is the observation that the conformation of the catalytic lysine K288 in the apo-enzyme does not fulfill the proximity requirement indispensable for the formation of the Schiff base with the cofactor PLP and for its participation in the necessary proton transfer steps. This inactive conformation of the catalytic lysine (backward conformation) can be spatially linked with the other three structural differences described in the literature in the transition from holo- to apo-enzyme (or *vice versa*), which cause a weakening of the monomer-monomer association and possibly dimer dissociation.

We envisioned that the conformational change of the catalytic lysine K288 upon PLP loss could be a slow spontaneous process leading to dissipation of the backbone strain associated with its conformation in the holo-enzyme (forward conformation). The newly solved PMP·*Cv*-ATA crystal complex confirmed that none of the known conformational rearrangements occur on the second time scale of crystal cryoprotection and, by extension, during the much faster time scale of catalytic turnover. A set of molecular dynamics simulations showed that, indeed, the rearrangement of K288 is a slow spontaneous process and that it can trigger the conformational changes occurring at the dimer interface expected to favor the dissociation of the *Cv*-ATA homodimer. In other words, the K288 rearrangement is the conformational switch triggering the cascade of conformational changes in the neighboring variability regions culminating in dimer dissociation. MD simulations and experimental results showed, in agreement with other reports^15^, that the coordination of a phosphate-containing species in the phosphate group binding cup located at the dimer interface can hinder the rearrangement of K288 to the backward conformation and contribute to improve the stability of the dimeric assembly. Nonetheless, as shown experimentally, the largest contribution to the thermal stability of the holo-*Cv*-ATA is the presence of the K288-PLP Schiff base linkage. Mutagenesis studies demonstrated that increasing the available space for K288 mobility leads to drastically improved holo-*Cv*-ATA thermal stability and significantly reduces the importance of the Schiff base for protein stability.

Due to the occurrence in the literature of similar PLP-dependent inactivation models verified using different methods for other non-transaminase PLP-dependent enzymes^17–19^ the validity of the lysine-dependent inactivation model presented in this paper might extend to other fold type I PLP-dependent enzymes of interest for biocatalytic applications, even outside of the transaminase class. This model, built on the case study of a known (*S*)-selective amino transaminase, might offer a new perspective to drive future non-trivial enzyme engineering approaches targeting a sub-group of fold type I PLP-dependent biocatalysts.

## Materials and Methods

### Enzyme cloning and generation of the K288A and Y322A mutants

The ω-transaminase gene from *C*. *violaceum* (GenBank accession WP_011135573) was previously inserted into pET28a(+) with a N-terminal His6-tag^33^. The plasmid sequence was verified (Eurofins MWG Operon, Ebersberg, Germany) before transformation into *E*. *coli* BL21(DE3). The enzyme variant K288A was generated according to Humble *et al*.^34^. The Y322A mutant was constructed by site-directed mutagenesis using the Phusion High-Fidelity DNA Polymerase from Thermo Fisher and following the Quick Change method with modifications according to Zheng *et al*.^35^. The primers used were (indicating the mutation in lower case):

CvTA_Y322A _fw: 5′-GTTTTACCgcgAGCGGTCACCCGGTGTG-3′

CvTA_Y322A _rv: 5′-CGCTcgcGGTAAAACCGTGGTTGAAGTCGC-3′

### Enzyme expression and purification for activity and stability assays

One colony of *Escherichia coli* BL21(DE3) containing WT *Cv*-ATA and variants in pET28a(+), was inoculated in LB medium (5 mL) supplemented with kanamycin (50 μg/ml). This pre-culture was grown overnight at 37 °C and 200 rpm and subsequently used to inoculate 400 mL of Terrific Broth (TB) medium supplemented with kanamycin (50 μg/ml). The culture was incubated at 200 rpm and 37 °C until OD_600_ reached 0.7–0.9. Expression was induced by supplementing isopropyl β-d-1-thiogalactopyranoside (IPTG, 1 mM) and carried out for 16 h at 20 °C. Cells were harvested by centrifugation at 8000 rpm for 30 min at 4 °C in a Beckman Coulter Avanti J-26XP centrifuge.

The collected cell pellet was re-suspended in binding buffer (50 mM NaH_2_PO_4_, 300 mM NaCl, 20 mM imidazole, pH 8.2) for disruption by sonication (6 min; Branson Sonifier 250) at 30 duty cycles % and with a 5–6 Micro tip limit. Cell debris was precipitated by centrifugation (4500 rpm for 30 min at 4 °C) before sterile-filtration (cut-off 0.45 μm) of the supernatant. Purification was performed on a Ni-NTA His-trap column, according to the IBA manufacturer´s manual and using NaH_2_PO_4_ (50 mM), NaCl (300 mM), imidazole (500 mM), pH 8.2, as elution buffer.

The apo and holo form of Cv-ATA were obtained according to Chen *et al*.^15^. The apo-*Cv*-ATA was obtained from the eluted samples after buffer-exchange to HEPES buffer (50 mM, pH 8.2) with no addition of PLP. The holo-*Cv*-ATA was obtained from the eluted samples after buffer-exchange to HEPES buffer (50 mM, pH 8.2, 5 mM PLP). After overnight incubation at at 4 °C followed by 1 h incubation at 37 °C, the excess PLP was removed in a desalting step performed using a PD-10 column. The abundance of apo/holo enzyme in the samples was calculated based on the ratio of Abs_415_/Abs_280_.

### Wild-type *Cv*-ATA protein expression and purification for crystallization

Wild-type holo-*Cv*-ATA preparations for crystallization purposes were produced starting from the same *E.coli* strain using similar protocols to those described for the preparations destined to activity and stability assays. The pre-cultivation and cultivation steps were performed according to the same procedures already described with the only exceptions of a cultivation inoculum of 10 ml pre-culture in 1 L TB medium supplemented with antibiotic. Expression was induced by addition of IPTG (0.4 mM) and performed for 24 h at 25 °C and 120 rpm agitation. Following cell harvest, pellets were resuspended in lysis buffer (50 mM HEPES pH 7.4, 500 mM NaCl, 10 mM imidazole, 0.1 mM phenylmethanesulfonyl fluoride) and sonicated on ice. After debris sedimentation (30 000 xg at 4 °C for 20 min), the soluble fraction was loaded onto a pre-equilibrated HisTrap FF crude column (5 ml, GE Healthcare). The target protein was eluted in a linear gradient to 500 mM imidazole in 20 min at a flow rate of 5 ml/min. The fractions containing the target enzyme were merged and concentrated to 2.2 ml using an Amicon Ultra Centrifugal Filter Unit (Merck, cut-off 30 kDa) prior to addition of PLP (0.6 mg) and further incubation at 4 °C in the dark overnight. Multimers of the target and excess of PLP were removed in one step gel filtration on a HiLoad 16/60 Superdex 200 prep grade (GE Healthcare). The dimeric protein was eluted in HEPES pH 8.4 (50 mM), concentrated to 13.7 mg/ml and flash frozen.

### Enzyme activity assays

A model transamination reaction was performed to assay enzyme activity. The reaction mixture (1 mL) generally composed of 10 μg of enzyme, (*S*)-1-phenylethylamine (2.5 mM) and pyruvate (2.5 mM) in 50 mM HEPES pH 8.2. After gentle inversion of the reaction mixture in the cuvette, the formation of acetophenone was dynamically monitored at 245 nm on a Cary 50 UV–vis Spectrophotometer.

An incubation of 0.5 mg/mL of purified enzyme in HEPES buffer (50 mM, pH 8.2) at 20 °C was used to monitor enzyme residual activity. When indicated, the enzyme solution was incubated with the designated additive. Residual activity was determined by transferring the enzyme samples at specific time intervals into the assay solution to perform the model amine transamination reaction with pyruvate and (*S*)-1-phenylethylamine.

### Experimental determination of the role of the K288-PLP Schiff base

The role of the Schiff base linkage in *Cv*-ATA stability was evaluated by spectra measurement, activity assay and DSF.

Protein aliquots stored at −80 °C were quickly thawed in the hand and placed on ice in the dark. Protein concentrations were measured in triplicates on a Nanodrop (Thermo Fisher) using the E 0.1% values calculated in ProtParam (ExPASy server)^36^ from the protein sequences complete of hexahistidine tags (E 0.1%_(Wild-type)_ = 1.560; E 0.1%_(Y322A)_ = 1.509).

UV-Vis spectra were measured starting from sample mixtures (100 μl) stored at 4 °C in the dark for 4 hours containing protein (5 mg/ml) in HEPES pH 8.2 (50 mM) and, where indicated, either NaCNBH_3_ (20 mM) for the Schiff base reduction or L-alanine (20 mM) for the conversion of PLP to PMP. Controls contained NaCNBH_3_ or L-alanine in buffer or just buffer. At the end of the incubation the samples were transferred in a UV Flat Bottom Microtiter plate (Thermo Fisher). Spectra were measured in the range 270–650 nm at 2 nm intervals in a Clariostar plate reader (BMG Labtech). The spectra were compared after having been normalized on the value of Abs_280_.

The residual specific activity of the samples was then evaluated by UV-Vis spectra recording activity in the acetophenone assay. The substrate solution (995 μl) containing (*S*)-1-phenylethylamine (5 mM) and sodium pyruvate (5 mM) was mixed in an UV-clear cuvette with 5 μl of the enzyme sample^37^. After mixing the assay solution by inversion, the increase of Abs_245_ was measured over time in a Cary 3000 spectrophotometer. The slope of the linear function fitting the points collected during the first minute was used to calculate the specific value of enzyme activity expressed in U/mg.

DSF measurements^24^ were performed on a CFX96 Real-Time PCR Detection System and C1000 Thermal Cycler at 569 nm after 4 h incubation at 4 °C of the protein mixtures (20 μL, 0.5 mg/ml enzyme, 3.75 × SYPRO Orange protein gel stain, 50 mM HEPES pH 8.2 and PLP or substrates supplementation when indicated). When present, the concentration of the reducing agent NaCNBH_3_^17^ or amino donor L-alanine was 20 mM. The temperature range was set to 25–95 °C, with a constantly increasing rate of 1 °C per min. The *T*_m_ value was determined by fitting the original data to the Boltzmann equation in GraphPad Prism 6.0.

### Wild-type *Cv*-ATA crystallization and cryoprotection

The holo-*Cv*-ATA aliquots were thawed quickly in the hand and the protein concentrations were measured on a DeNoviz nanophotometer (E 0.1% = 1.560). Crystallization drops of 0.4 μl volume (0.2 μl reservoir + 0.2 μl protein 7.5 mg/ml) were dispensed with the aid of a TTP Labtech Mosquito in MRC 3-well plates (Molecular Dimensions Ltd.) for sitting drop vapor diffusion crystallization. Single crystals grew to their maximum size in HEPES pH 7.5 (100 mM), NaCl (350 mM), PEG 4000 (22.5% *w*/*v*), PLP (0.1 mM) in 0.4 μl crystallization drops (0.2 μl reservoir + 0.2 μl protein 7.5 mg/ml) left at room temperature in the dark for about one week. Prior to flash freezing the crystals were cryoprotected in HEPES pH 7.5 (100 mM), NaCl (350 mM), PEG 4000 (22.5% *w*/*v*), PLP (0.1 mM), glycerol (20% *v*/*v*) and 1-(*S*)-phenylethylamine (20 mM) (TCI Chemicals).

### Data collection and structure determination

X-ray diffraction data was collected with a PILATUS 6 M detector at BL 14.1 of the BESSY synchrotron^38^, HZB, Berlin, Germany at 100 K and λ = 0.918 Å. Passes of 0.1° were used in the collection of two complete 360° datasets differing by 20° in the value of the goniostat κ angle. After initial data processing with XDSAPP^39^ the two datasets were scaled and merged together in Aimless^40,41^ (CCP4 program suite, v 7.0.015, Aimless v 0.5.27) to improve the low completeness characteristic of space group P1. Data collection and data quality statistics are reported in Table S2. Initial phases were calculated in Phaser-MR^42^ (CCP4 program suite, v 7.0.015, Phaser-MR v 2.6.1) using the solvent- and PLP-deprived structure of the *Cv*-ATA (PDB 4A6T) as search model. Phases were progressively improved through iterated cycles of automated and manual real space refinement in Refmac5^43^ (v. 5.8.0238) and Coot (v 0.8.9.1)^44^. PMP and water molecules were manually added in Coot. The final model at 1.67 Å resolution was validated using Procheck^45,46^.

### Structure analysis

Structures were analyzed and compared using Coot (v 0.8.9.1)^44^, MacPyMOL (v 1.8.4.1) and YASARA (v 17.1.28 for the docking or 18.4.24 for the MD simulation)^47^. Procheck^45^ (CCP4 program suite v 7.0.015) was used to assess structure quality. Ramachandran plots were calculated using the Dynarama tool implemented in Coot (v. 0.8.9.2)^44^.

### MD simulations

All molecular dynamics (MD) calculations were performed using the md_run.mcr macro included in the YASARA package (v 18.4.24)^47^ within a water-filled cubic 110.35 Å^3^ simulation cell generated around all protein atoms. Every starting *Cv*-ATA crystallographic structure was energy-minimized in an AMBER14 force field at the chosen simulation pH. Simulation pH and temperature were 8.2 and 373 K, respectively. Intramolecular forces were calculated every two simulation sub-steps, each of the duration of 1.25 fs. Simulation snapshots were automatically saved at 0.1 ns simulation intervals for a total of 40 ns. After conversion to pdb format the snapshots were compared in MacPyMOL (v 1.8.4.1).

### MD simulations on the *Cv*-ATA holo-monomer (PLP-depleted and complete of PLP)

The AB dimer complete of PLP, crystallographic waters and hydrogen atoms (pH 8.2) was energy-minimized. After the energy minimization step chain B was deleted from the simulation soup. The PLP molecule belonging to chain A was either removed or kept, depending on the experiment (either PLP-depleted or not).

### MD simulation on the N-terminal and PLP-depleted *Cv*-ATA holo-dimer

The AB dimer complete of PLP, crystallographic waters and hydrogen atoms (pH 8.2) was energy-minimized. The PLP molecules and the N-terminal domain (res. 5–35) were then removed from both chains.

### MD simulation on the N-terminal-depleted *Cv*-ATA holo-dimer in the presence of methyl phosphonate or PLP-desphosphate

The AB dimer complete of PLP, crystallographic waters and hydrogen atoms (pH 8.2) was energy-minimized. The N-terminal domain (res. 5–35) was deleted in both monomers. The cofactor molecule was cropped by manually removing either everything except the phosphate methyl-ester moiety or nothing but it.

### Docking

Docking experiments were performed in YASARA^47^ (v 17.1.28) using the macro dock_run.mcr at pH 8.2 in an AMBER14 force field in a simulation environment containing the AB dimer of the Cv-ATA structure (PDB ID: 4A6T) as receptor and the *in-silico* generated models of the binding molecules as ligands.

The hydrogen-complete protein receptor (pH 8.2) was energy minimized after a preliminary step of optimization targeting the orientation of the added hydrogen atoms. The rigid water- and PLP-depleted dimer was used as receptor in a 12 Å^2^ cubic simulation cell centered on the K288 Cα (chain A).

The PLP-gabaculine external aldimine ligand was generated and normalized in JLigand (v 1.0). The PLP-gabaculine external aldimine (Supplementary, Figure S10, panel a) was created by replacing the 4′-oxygen of a PLP molecule imported from the CCP4 library with the 2,3-dihydrobenzoic group of the gabaculine inhibitor. The final hydrogen-complete and normalized molecule was then used as ligand in the docking simulations.

The docking solutions were scored based on their automatically-generated dissociation constants and on the RMDS between the phosphate group of the docking solutions and that of the phosphate group of PLP in the 4A6T structure.

### Retrieval of fold type I structures and structure analysis

The RCSB PDB-wide structure search was performed submitting the holo-*Cv*-TA structure as query on the Dali server (http://ekhidna2.biocenter.helsinki.fi/dali/)^29^. The 2187 hits (Z-scores 72.2-11.9) were manually sorted to exclude repeated chains in multimeric assemblies, enzyme/substrate and enzyme/inhibitor complexes, mutants and ancient enzyme reconstructions. The initial working set of 22 structures was selected by searching the PDB for different enzyme forms of each candidate hit. Sequences and enzyme function data were collected from the RCS PDB (https://www.rcsb.org)^48^, UniprotKB (https://www.uniprot.org)^49^, BRENDA (https://www.brenda-enzymes.org)^50^ and BioCyc (https://biocyc.org)^51^ databases. The 3D structures of these enzymes were compared to the structures of the *Cv*-TA dimer (PDB IDs: holo 4A6T chains AB, apo 4A6R) using Pymol (v. 1.8.4.1). Monomeric structures were aligned to *Cv*-TA chain A (apo or holo). When multiple dimers were present in the crystallographic unit, as well as in the case of tetrameric assemblies, only the AB dimers were considered for comparison. The *Cv*-TA Y322 structural analogues were identified by inspecting the structural alignments. The alignment between *Cv*-TA and *Ht* was performed on YASARA^47^ using the MUSTANG structure alignment algorithm^52^. Sequences were compared by running a BLAST multiple sequence alignment with default settings using the sequence of the *Cv*-TA monomer as query^53^.

## Supplementary information

Supplementary information
