# Supplementary material for: Insight into the dimer dissociation process of the Chromobacterium violaceum (S)-selective amine transaminase
Source: Sci Rep. 2019 Nov 18;9:16946. doi: 10.1038/s41598-019-53177-3 (PMC6861513; doi:10.1038/s41598-019-53177-3)
Supplement: Supplementary file 1 — Supplementary information [file 41598_2019_53177_MOESM1_ESM.pdf]

Insight into the dimer dissociation process of the *Chromobacterium violaceum* (*S*)-selective amine transaminase

Federica Ruggieri[1,2], Jonatan C. Campillo-Brocal[1], Shan Chen[1], Maria S. Humble[3], Björn Walse[2], Derek T. Logan[2], Per Berglund*[1]

[1] Department of Industrial Biotechnology, KTH Royal Institute of Technology, AlbaNova University Center, SE-106 91 Stockholm, Sweden

[2] SARomics Biostructures AB, Medicon Village, SE-223 81 Lund, Sweden

[3] Pharem Biotech AB, Biovation Park, SE-151 36 Södertälje, Sweden

* perbe@kth.se

Supplementary Information

Figure S1-S15
Scheme S1
Table S1-S4

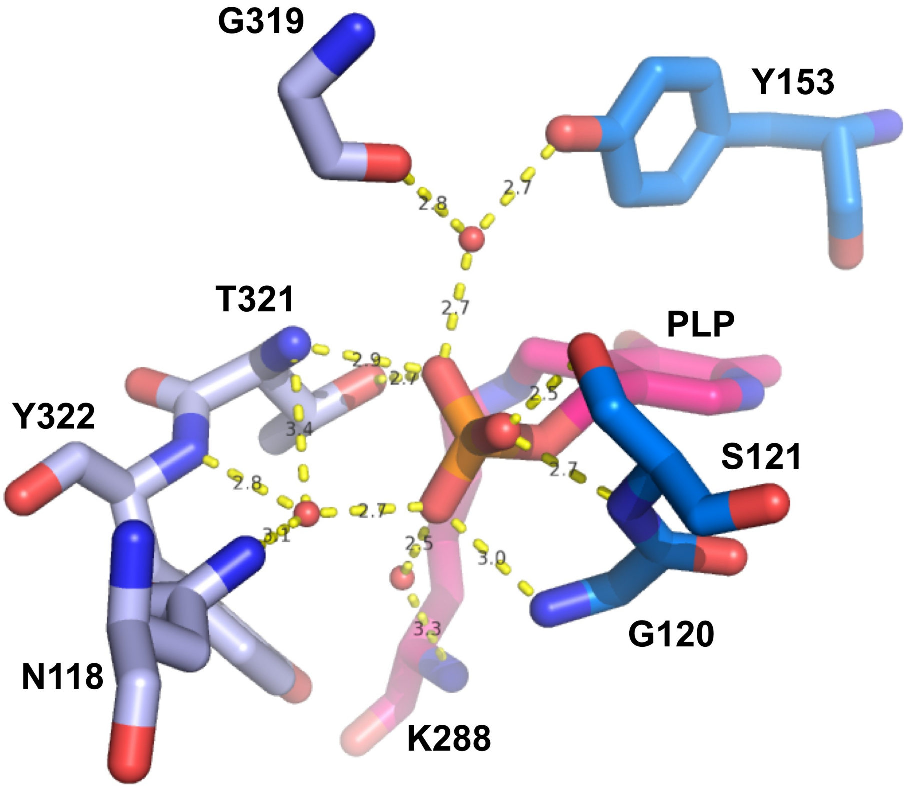

Figure S1. Hydrogen-bonding network in the phosphate group binding cup (PGBC) of the wild-type *Cv*-ATA. The 14 hydrogen bonds spanning across the PGBC involve the phosphate group of PLP either directly or through the mediation of 3 water molecules. Residues from both dimer subunits are involved in the network of interactions. Residues belonging to different chains are represented in different tones of light blue, the K288-PLP is represented in pink (PDB ID 4A6T)

Table S1. Data quality of the *Cv*-TA crystal structures mentioned in this study. The letters reported in the "electron densities" column are cross-references to the panels in Figure S2.

Cv structure (PDB ID)	Resolution (Å)	R_{work}/R_{free} (%)	Electron densities	References
holo-*Cv*-TA (4A6T)	1.80	16.5 / 20.2	**a**	[1]
apo-*Cv*-TA (4A6R)	1.35	13.4 / 16.3	**b**	[1]
GABA·*Cv*-TA (4BA5)	1.76	17.4 / 23.3	**c**	[2]

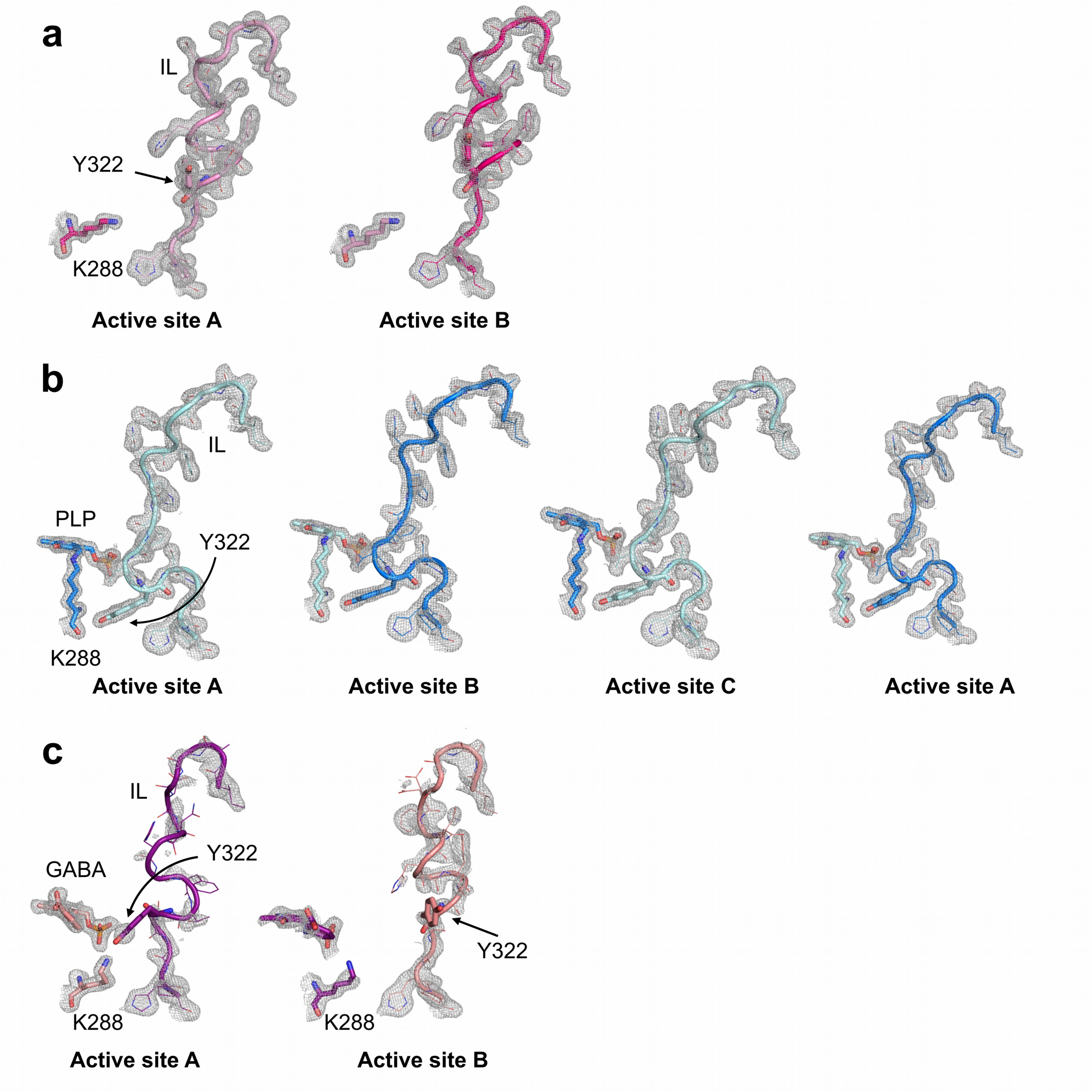

Figure S2. Experimental electron densities (2Fo-Fc map, contouring = 1σ) for the structure segments discussed in this study (K288, Y322 and interfacial loop IL) in the known apo-*Cv*-TA (panel a), holo-*Cv*-TA (panel b) and GABA·*Cv*-TA (panel c) crystal structures (PDB IDs: 4A6R, 4A6T and 4BA5, respectively). For each structure, densities are shown for all chains contained in the asymmetric unit, with "active site A" referring to the active site containing the catalytic lysine K288 contributed by chain A etc. The side chains of K288 and Y322 as well as the ligands PLP and PLP-gabaculine are represented as sticks. All other residues belonging to the IL are represented as lines. Resolutions and R-factors for each structure are reported in Table S1.

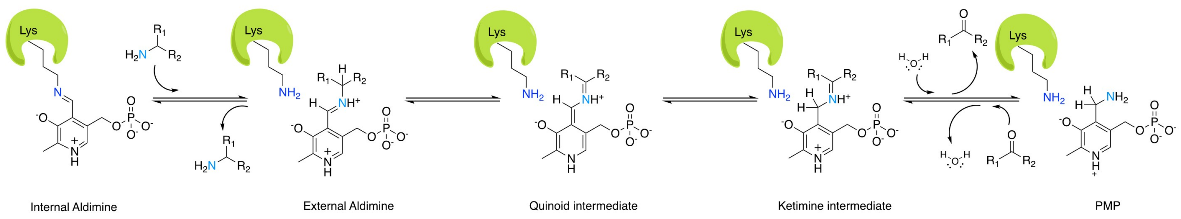

Scheme S1. General simplified transaminase reaction mechanism. The first transamination half-reaction (from E-PLP to E·PMP) is shown from left to right, the second half-reaction (from E·PMP to E-PLP) is shown from right to left.

Table S2. Quality statistics about data collection, data processing and model generation and refinement. Values referring to the highest resolution shell (1.67 – 1.713 Å) are shown in parenthesis.

**Data collection**	
Space group	P1
Cell dimensions	
a, *b*, *c* (Å)	60.91, 61.97, 118.81
α, β, γ (°)	75.07, 81.31, 75.30
Resolution (Å)	48.09 - 1.67
Rmerge (%)	9.1 (64.6)
Rmeas (%)	11.1 (81.0)
Rpim (%)	6.1 (48.0)
CC1/2 (%)	99.3 (65.4)
I/σI	5.3 (1.71)
Completeness (%)	91.9 (87.0)
Redundancy	2.4 (2.3)
**Refinement**	
Resolution (Å)	1.67
No. reflections	163935
R_{work}/R_{free} (%)	16.1 (26.4) / 18.5 (29.0)
No. atoms	
Protein	14310
Ligand	98
Number of water molecules	481
B-factors	
Protein	27.6
Ligand	20.4
Water	25.4
R.m.s. deviations	
Bond lengths (Å)	0.014
Bond angles (°)	1.756

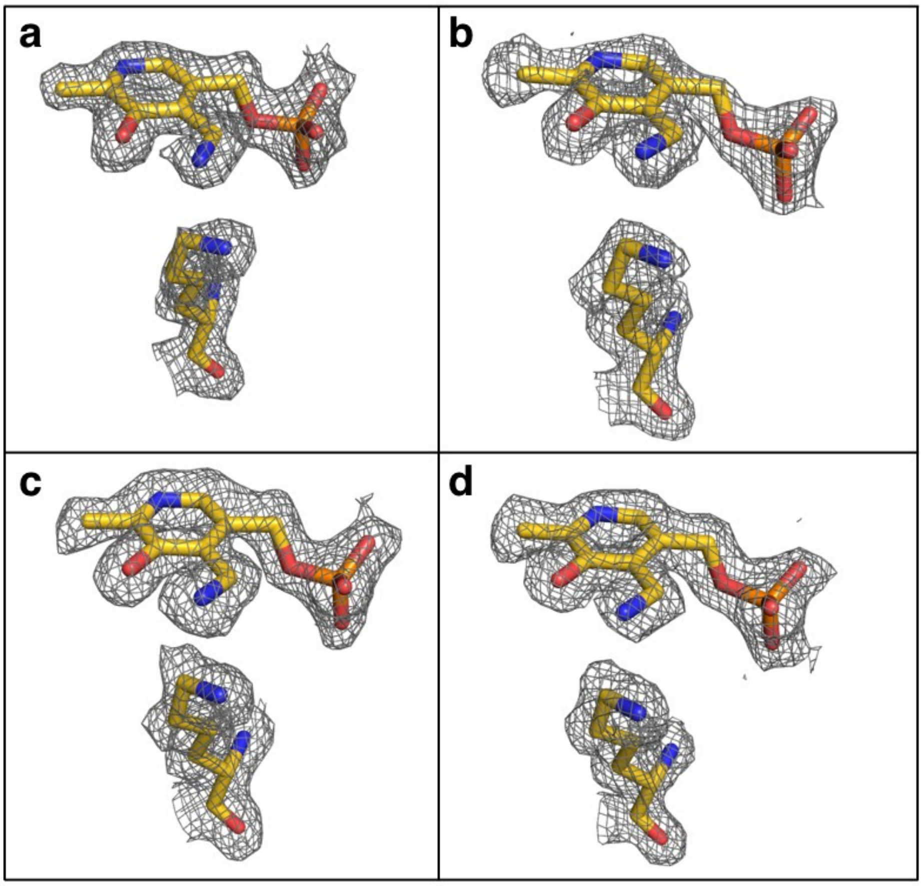

Figure S3. Discontinuous experimental electron density between the catalytic lysine K288 side-chain and the cofactor moiety (2Fo-Fc map countered at 1.0 σ). Panel a: chain A; panel b: chain B; panel c: chain C, panel d: chain D. (PDB ID 6S4G)

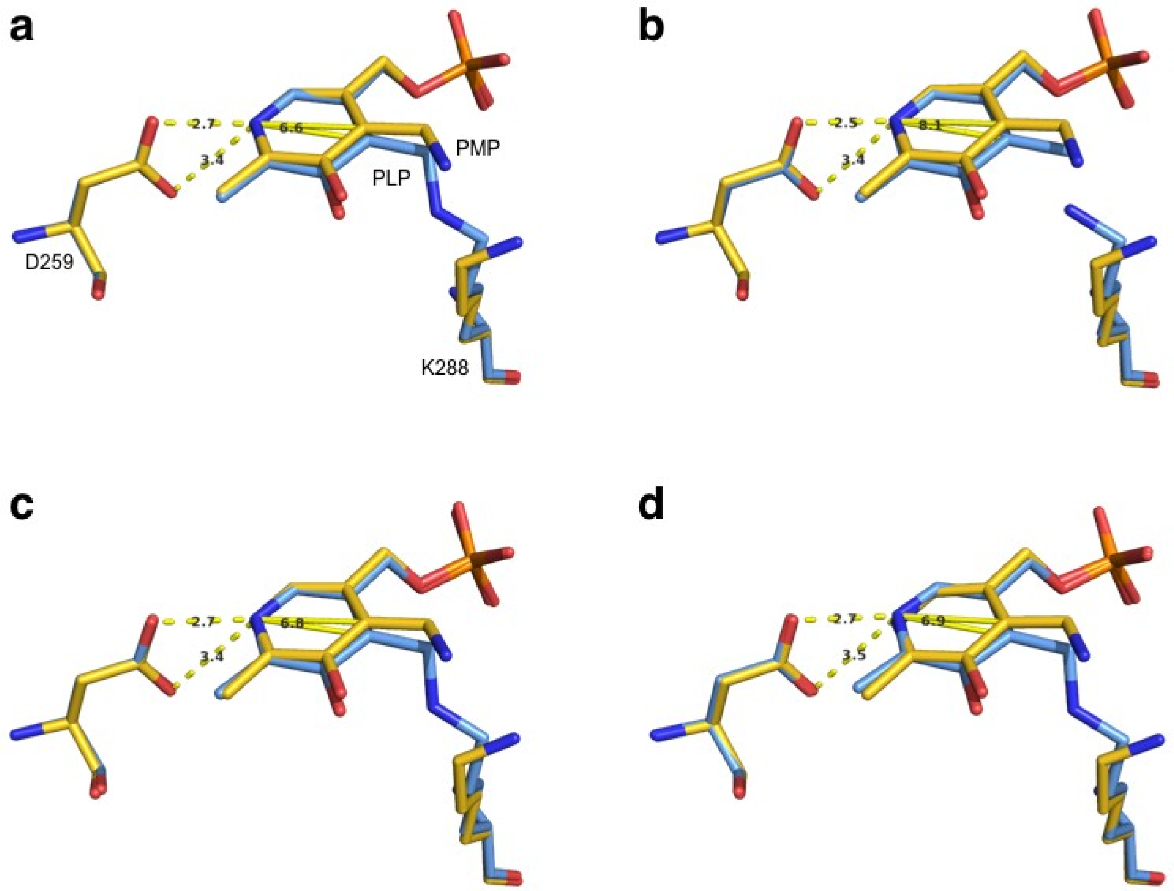

Figure S4. Tilt-twist rearrangement of the cofactor upon conversion to PMP. The four protein chains show slightly different values for the combined tilt and twist reorganization of the cofactor, represented, for comparison, both as participating in the Schiff-base with K288 (in light blue) and as PMP (in yellow). The cofactor ring reorientation is hinged on the pyridine nitrogen of the cofactor ring, which is held in position by the bidentate polar interaction with the side-chain of D259 (panel a: chain A, panel b: chain B, panel c: chain C, panel d: chain D).

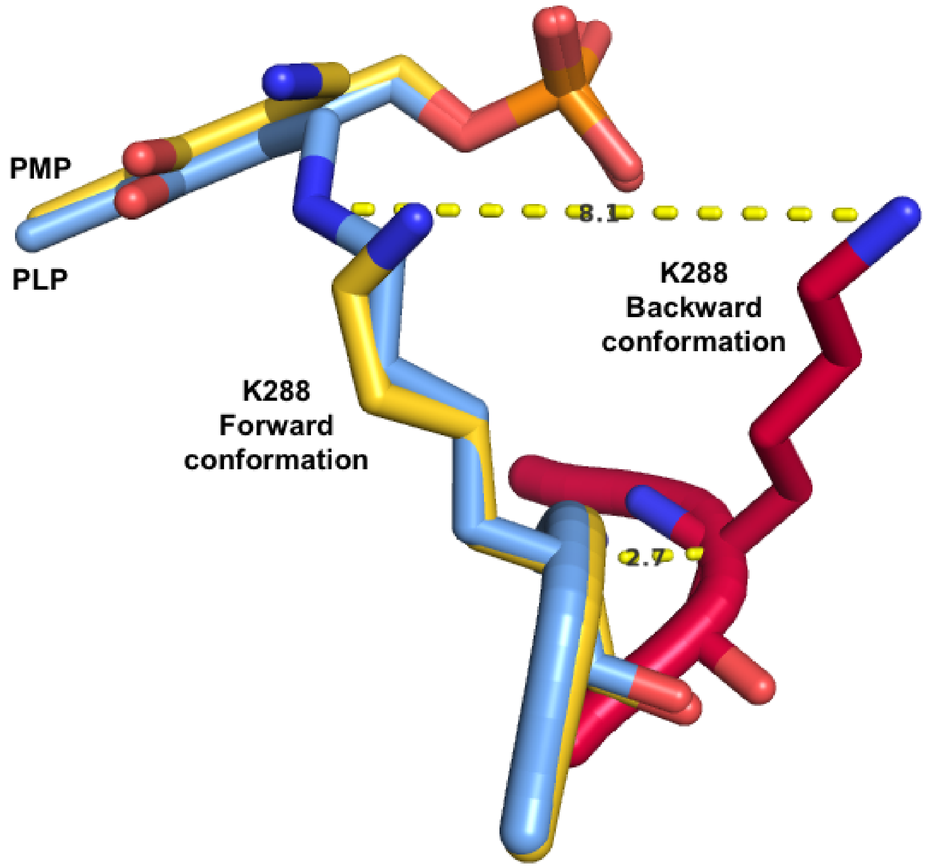

Figure S5. Lysine K288 “forward” and “backward” conformations compared by superposition of apo-*Cv*-ATA (PDB 4A6R, chain A, in pink), holo-*Cv*-ATA (PDB 4A6T, chain A, in light blue), PMP·*Cv*-ATA (PDB 6S4G, chain A, in yellow). The apo-*Cv*-ATA model presents the K288 backward conformation, the holo- and PMP·*Cv*-ATA show the forward K288 conformation, in which the NZ group is located in close proximity to the 4’ group of the cofactor. The two conformations are related to each other through a rearrangement of the backbone that results in a broad swing of the K288 side-chain. The K288 rearrangement causes a 8.1 Å linear displacement of the NZ group and a 2.7 Å linear displacement of the Cα-carbon.

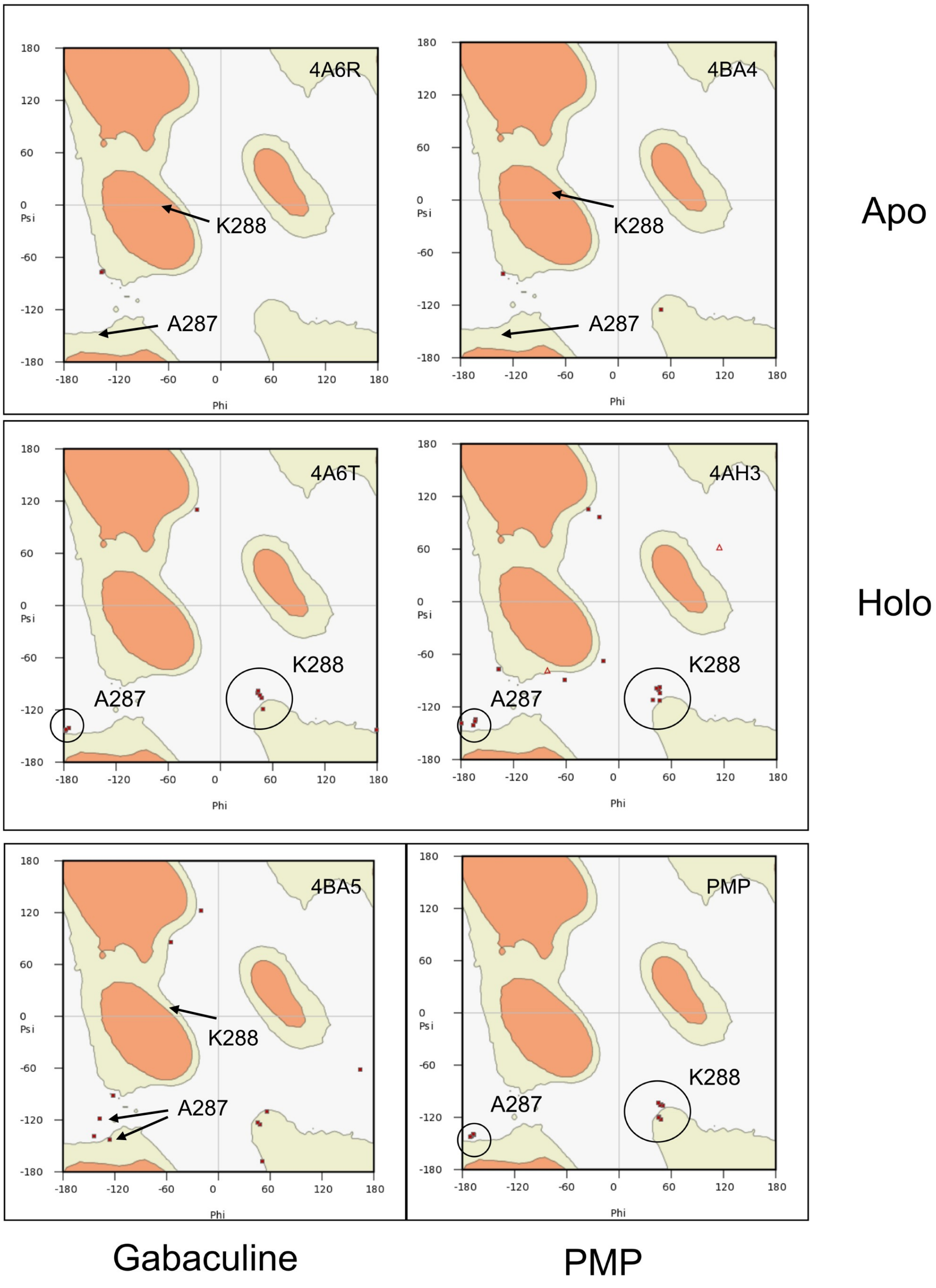

Figure S6. Plots showing the distribution of Ramachandran outliers in all deposited *Cv*-ATA crystal structures. Outliers conformations of A287 and K288 are encircled in black. Ramachandran outliers that are not encircled correspond to other residues. The location in the plot of A287 and K288 in the apo enzyme and in the gabaculine complex are shown by arrows.

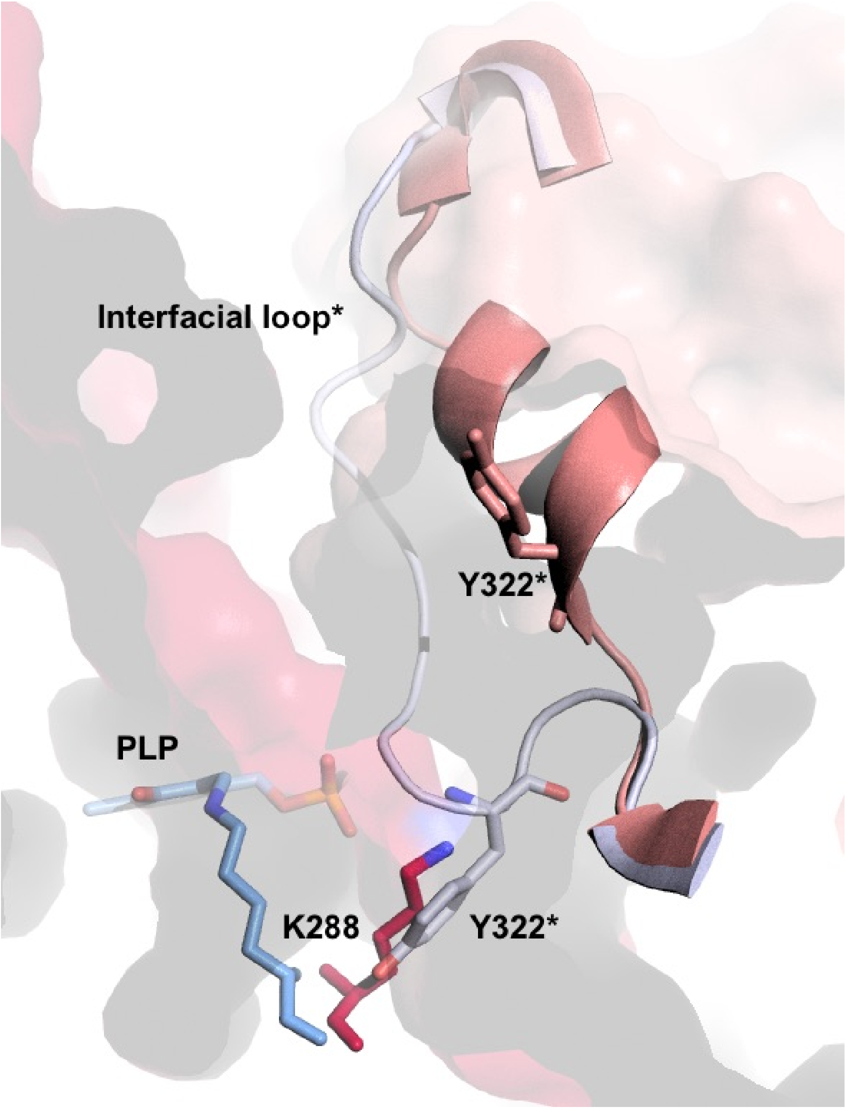

Figure S7. The relaxation of the K288 to the backward conformation as conformational switch. In the holo-*Cv*-ATA the K288 is found in the forward conformation (PDB ID 4A6T, chain A represented in a darker blue, chain B represented in a lighter blue. PLP and K288 are represented as sticks). Its side-chain is stretched inside the active site, where it participates to the covalent Schiff-base with the cofactor PLP. The interfacial loop* is stretched inside the active site, kept in position by the hydrogen bonds established between the phosphate group of the cofactor and the residues T321* and 322*. In the apo-*Cv*-ATA the K288 is found in the backward conformation (PDB ID 4A6R, chain A represented in a darker pink, chain B represented in lighter pink, Y322* is represented as sticks, the interfacial loop* is represented as cartoon). The relaxation of the K288 from the forward to the backward conformation is incompatible with the extended conformation of the interfacial loop* inside the active site. To avoid the steric clash with the side chain of Y322* the interfacial loop* recoils away from the active site cavity.

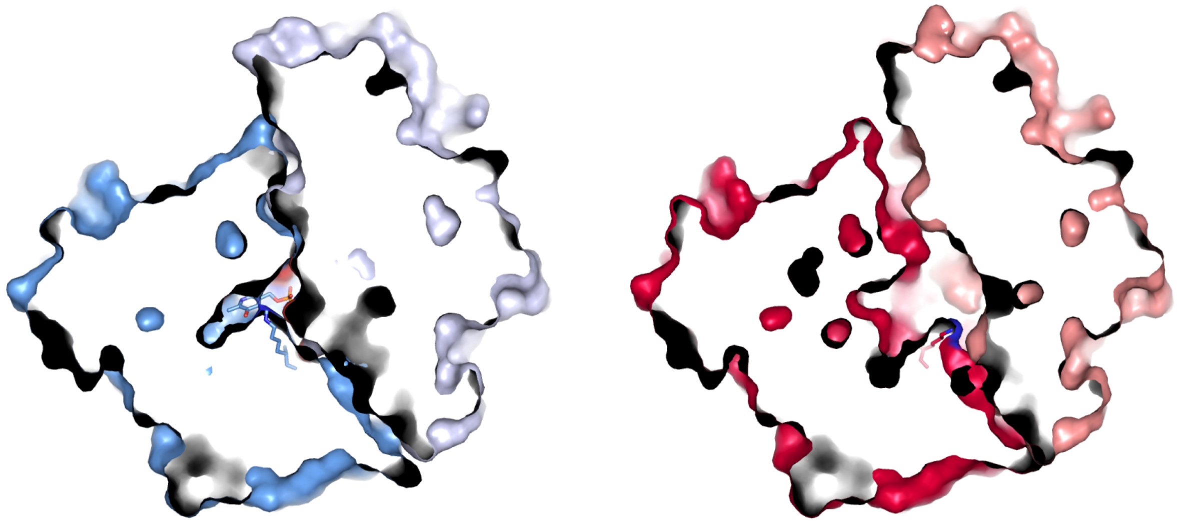

Figure S8. The weakening of the *Cv*-ATA dimer interface upon transition from holo- to apo- form. In the holo-*Cv*-ATA (on the left) the forward conformation of the K288 and the extension of the interfacial loop* create a tight dimer interface (PDB ID 4A6T, chain A represented in a darker blue, chain B represented in a lighter blue. PLP and K288 are represented as sticks). In the apo-*Cv*-ATA (on the right) the backward conformation of the K288 broadens the active site cavity. The recoiling of the interfacial loop opens a deep cleft spanning across the dimer interface. When these two rearrangements occur in both active sites, two wide hollow cavities weaken the core of the dimer while the two deep clefts, opening from opposite sides of the interface, separate the monomers. The dissociation of the dimer is further promoted by the displacement of the N-terminal loop (PDB ID 4A6R, chain A represented in pink, chain B represented in lighter pink. K288 is represented as sticks).

Table S3. Initial crystallographic B-factors for the structural elements lining the two active sites in the MD simulation of the PLP-depleted N-ter-depleted *Cv*-ATA homo-dimer. Structural elements belonging to the same active site are color-coded. For completeness, the B-factor values for the chains not used in the simulation (chains C and D) are also reported.

Chain ID	B-factor K288	B-factor Y322	Overall average B-factor
A	26.1	28.6	29.9
B	29.1	22.7	25.8
C	16.3	19.5	20.5
D	22.1	11.9	27.7

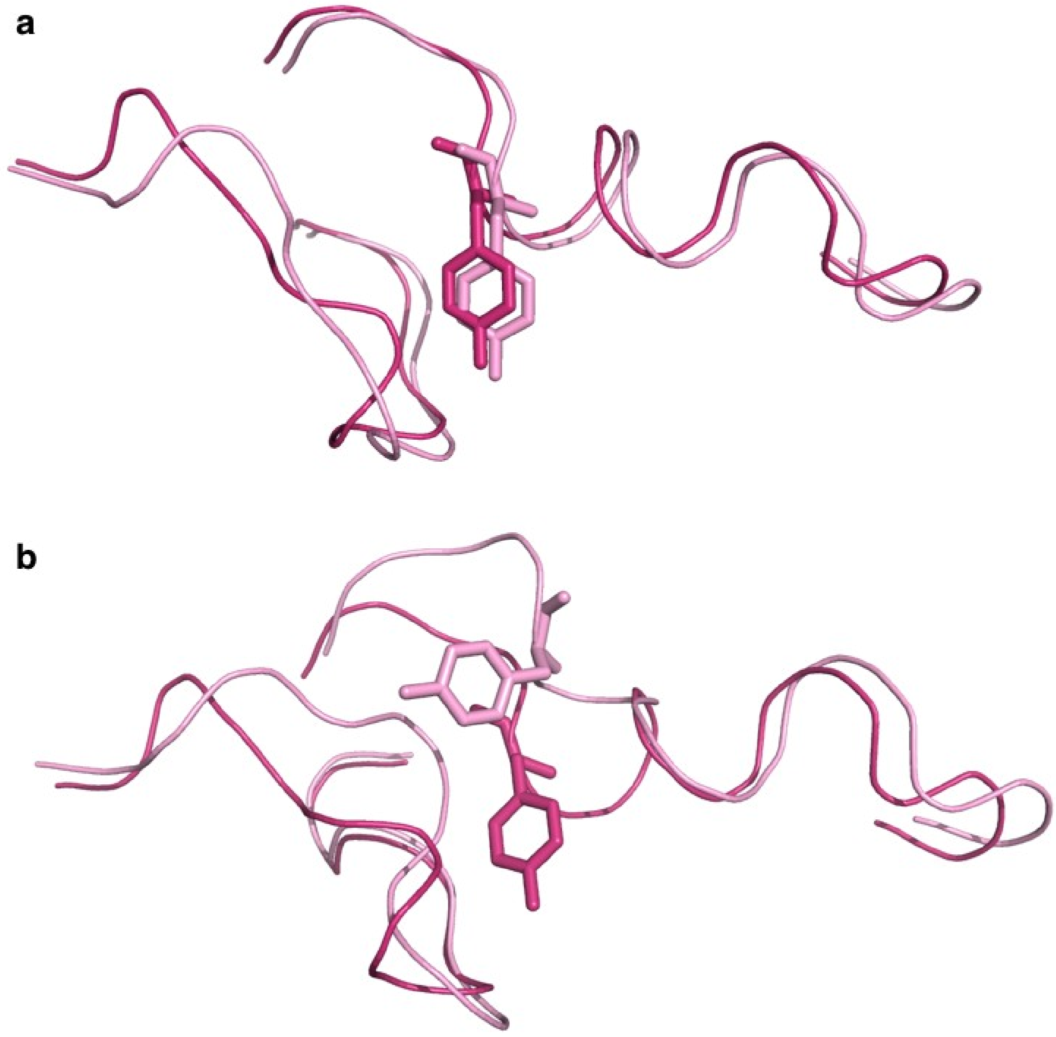

Figure S9. Comparison between the outer loops and interfacial loops in the GABA·*Cv*-ATA (PDB ID 4BA5, in light pink) and the apo-*Cv*-ATA (PDB ID 4A6R, in darker pink). The conformation of the interfacial loop in chain A of the complex (panel a) is identical to the recoiled conformation characteristic of the apo-structure. In particular, the side-chain of Y322 (shown as sticks) is completely reoriented. In chain B (panel b), most of the recoiling of the backbone has occurred. The region around Y322 is in an intermediate conformation between those observed in the holo- and in the apo-*Cv*-ATA.

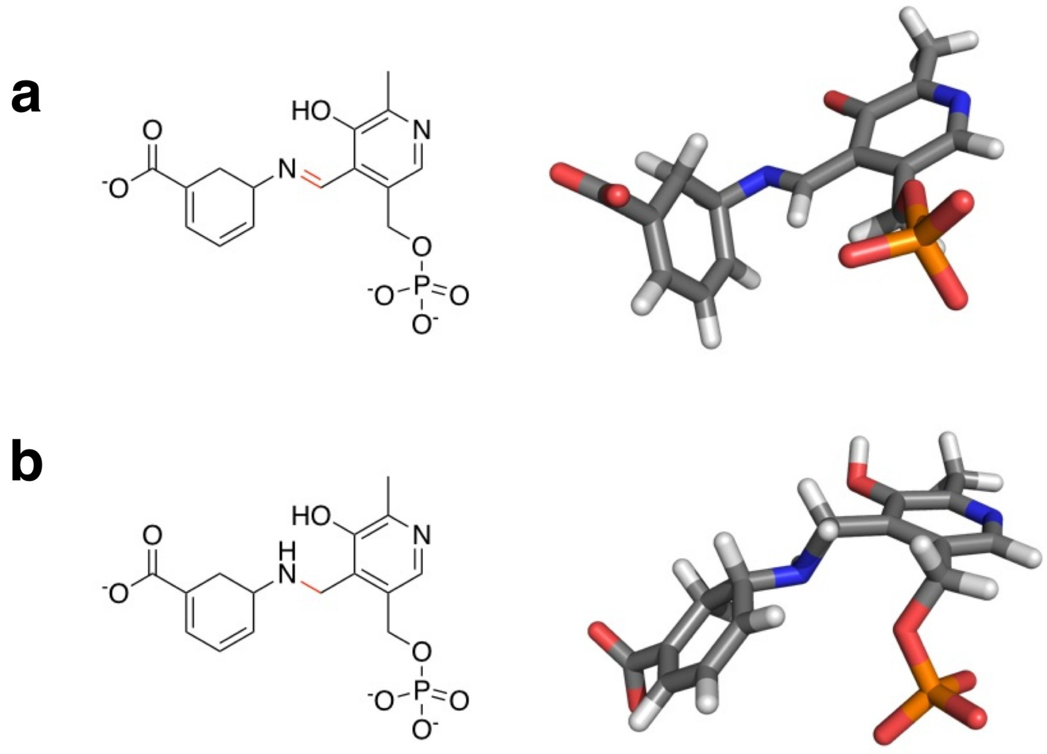

Figure S10. Comparison between the 3D conformation of the real expected PLP-gabaculine transition state intermediate (panel a) and PLP-gabaculine dead-end inhibitory adduct (panel b). The spontaneous elimination reaction occurring during the formation of the adduct affects the bond order, and hence the geometry and the rotational freedom, of the bond highlighted in red in the left-hand side of both panels.

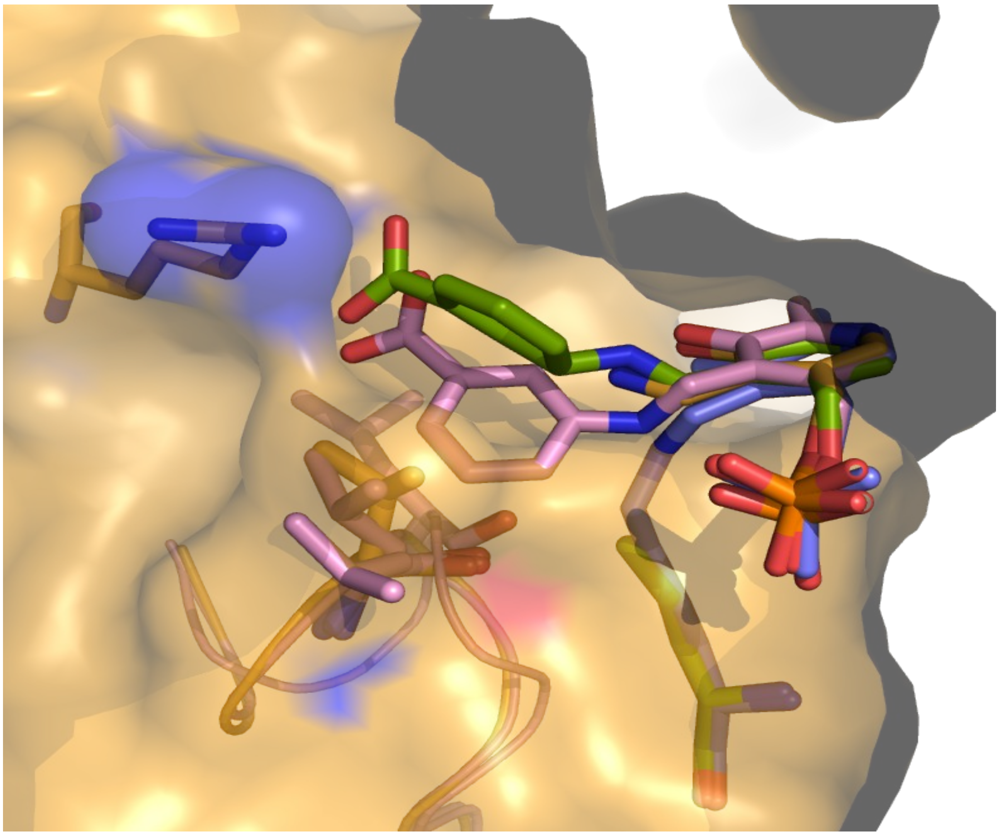

Figure S11. Comparison of the available *Cv*-ATA complexes. The holo-*Cv*-ATA (PDB ID 4A6T) is shown in purple, the PMP·*Cv*-ATA (PDB ID 6S4G) is shown in gold, the top scoring docking ligand is shown in green, the GABA·*Cv*-ATA (PDB ID 4BA5) is shown in pink. The gabaculine-PLP adduct and the gabaculine-PLP transition state (shown as sticks) are characterized by different 3D conformations. The gabaculine-PLP transition state possesses the correct 3D conformation to bind in the active site of the holo-*Cv*-ATA without requiring reorientation of L59 (shown as sticks). This orientation would also establish better contacts with the guanidinium group of the flipping arginine R416 (shown as sticks).

Table S4. Subset of 3D structures selected from the 3D search in the RCSB PDB

Organism	Code	Class [a]	PDB *Apo*[b]	PBD *Holo*[b]	Structural Identity %	Interfacial loop residue
Chromobacterium violaceum WT	*Cv*	2.6.1.62	4A6R (**D**)	4A6T (**D+D**)	(100)	Y
Vibrio fluvialis	*Vf*	2.6.1.2	5ZTX (**D**)	4E3R (**T**)	37	A
Lactobacillus buchneri	*Lb*	5.1.1.21	5LL2 (**T**)	5LL3 (**T**)	30	T
Pseudomonas jersenii	*Pj*	2.6.1.x	6G4B (**D**)	6G4D (**D**)	39	G
Halomonas elongata	*He*	2.6.1.x	-	6GWI (**D**)	57	Y
Mesorhizobium loti	*Ml*	2.6.1.1	-	3GJU (**M**)	37	Y
Virgibacillus sp.	*Vs*	2.6.1.x	-	6FYQ (**M**)	37	Y
Paracoccus denitrificans	*Pd*	2.6.1.-	-	4GRX (**D+D**)	37	A
Silicibacter sp.	*Ssp*	2.6.1.x	-	3FCR (**M**)	36	Y
Uncultivated *Pseudomonas*	*P*	2.6.1.x	-	5LH9 (**T**)	34	Y
Rhodobacter sphaeroides	*Rs*	2.6.1.62	-	3I5T (**D**)	33	Y
Pseudomonas aeruginosa	*Pa*	2.6.1.18	-	4BQ0 (**T**)	31	Y
Salmonella typhimurium	*St*	2.6.1.11	-	2PB0 (**D**)	25	Y
Hydrogenimonas thermophilia	*Ht*	2.8.1.7	-	5ZSP (**D**)	12	-
Pseudomonas sp.	*Ps*	?	5TI8 (**D**)	-	59	Y
Pseudomonas putida	*Pp*	2.6.1.82	6HX9 (**D**)	-	55	Y
Silicibacter pomeroyi	*Sp*	2.6.1.1/62	3HMU (**D**)	-	53	Y
Bacillus anthracis	*Ba*	2.6.1.62	3N5M (**T**)	-	29	F

[a] Completed, when possible, according to search in the BioCyc database
[b] Four-letter PDB code. Crystallographic quaternary structure in brackets: **M** monomer; **D** dimer: **D+D** dimer of dimers: **T** tetramers.

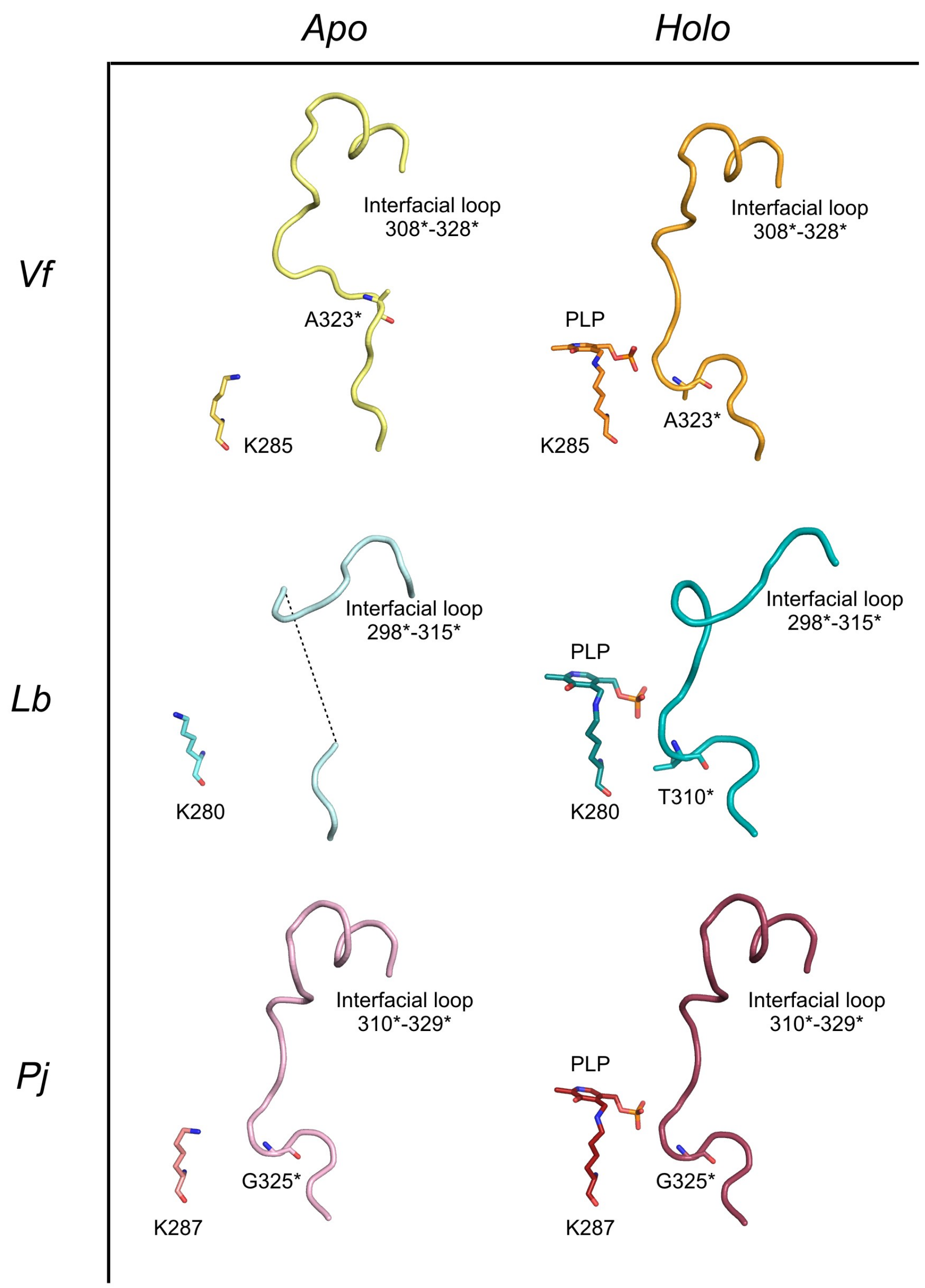

Figure S12. Structural analysis of the interface organization in the available *Vf* (*Vibrio fluvialis* transaminase), *Lb* (*Lactobacillus buchneri* isoleucine 2-epimerase) and *Pj* (*Pseudomonas jersenii* transaminase) apo- (on the left) and holo- (on the right) crystal structures. In each structure the catalytic lysine, the PLP (when present) and the structural analogue to the *Cv*-TA Y322 are shown as sticks, while the backbone of the region corresponding to the interfacial loop is shown as tube. (PDB IDs: apo-*Vf* 5ZTX; holo-*Vf* 4E3R; apo-*Lb* 5LL2; holo-*Lb* 5LL3; apo-*Pj* 6G4B; holo-*Pj* 6G4D).

a

Sequences producing significant alignments:

Description	Max Score	Total Score	Query cover	E Value	Per. Ident
Pseudomonas sp	579	579	97%	0.0	59.78
Pseudomonas putida	549	549	96%	0.0	57.60
Halomonas elongata	549	549	97%	0.0	57.11
Silicibacter pomeroyi	500	500	98%	6e-179	53.42
Pseudomonas jersenii	343	343	97%	1e-117	39.69
Mesorhizobium loti	322	322	94%	3e-109	39.33
Silicibacter sp	313	313	97%	6e-106	36.76
Vibrio fluvialis	310	310	95%	2e-104	38.75
Virgibacillus sp	308	308	97%	2e-103	36.80
Paracoccus denitrificans	302	302	95%	3e-101	38.31
Uncultivated Pseudomonas	290	308	95%	1e-96	35.47
Rhodobacter sphaeroides	274	274	94%	2e-90	34.10
Bacillus anthracis	238	238	93%	8e-77	29.61
Pseudomonas aeruginosa	223	223	83%	4e-71	33.76
Lactobacillus buchneri	178	178	90%	6e-54	30.90
Salmonella typhimurium	151	151	87%	2e-44	28.99

b

Query range : 361 to 420

```
Chromobacterium violaceum      315  --D----FN----HGFTYSGHPVCAAVAHANVAALRDEGIVQRVKDDIGPYMQKRWRETF  364
Pseudomonas sp.                327  --E----FY----HGFTYSGHPVAAAVALENIRILREEKIVETVKAETAPYLQKRWQE-L  375
Pseudomonas putida             316  --D----FN----HGFTYSGHPVAAAVGLENLRILRDEQIVEKARTEAAPYLQKRLRE-L  364
Halomonas elongata             313  --E----FF----HGFTYSGHPTCAAVALKNLELLEAEGVVDRVRDDLGPYLAERWA---  359
Silicibacter pomeroyi          318  --E----FN----HGYTYSGHPVAAAVALENLRILEEENILDHVRNVAAPYLKEKW-EAL  366
Pseudomonas jersenii           318  --A----LG----HGFTGSGHPVATAVALENLKIIEEESLVEHAAQ-----MGQLLRSGL  362
Mesorhizobium loti             317  --DKLGSLG----HGWTYSAHPICVAAGVANLELIDEMDLVTNA-GETGAYFRAELAKAV  369
Silicibacter sp.               316  --D----ENGPIGHGWTYSAHPIGAAAGVANLKLLDELNLVSNAG-EVGAYLNATMAEAL  368
Vibrio fluvialis               330  --E----FP----HGFTASGHPVGCAIALKAIDVVMNEGLAENVR-RLAPRFEERLKHIA  378
Virgibacillus sp.              328  --T----LF----HGFTYSGHPTAAAVALKNIAIIKEERLVENSK-RMGDALLHGLKKVK  376
Paracoccus denitrificans       328  --E----FP----HGFTASGHPVGCAIALKAIDVVMNEGLAENVR-RLAPRFEAGLKRIA  376
Uncultivated Pseudomonas       313  --G----FSHMIMHGYTYSGHPTACAAALAVLDIVEAEDLPGNAA-KVGAQLLEQLQPLV  365
Rhodobacter sphaeroides        322  --W----FT----NGYTYSNQPVACAAALANIELMEREGIVDQAR-EMADYFAAAL-ASL  369
Bacillus anthracis             311  --EYEF-FR----HINTFGGNPAACALALKNLEIIENENLIER-SAQMGSLLLEQLKEEI  362
Pseudomonas aeruginosa         319  AVE----FS----HGYTYSAHPVACAAGLAALDILARDNLVQQ-SAELAPHFEKGL-HGL  368
Lactobacillus buchneri         326  -------------HLFTTAGNPVCSAASLATLDVIEYEGLVEKSATD-GAYAKQRFLEMQ  371
Salmonella typhimurium         285  --------S----HGSTYGGNPLACAVAGAAFDIINTPEVLQGIHTKRQQFVQ-HLQAID  331
```

Figure S13. Multiple sequence alignment performed using the working set of enzymes and the *Cv*-TA sequence as query. Panel a shows the overall alignment scores. Panel B shows the multiple sequence alignment for the sequence segment containing the structural analogues to the *Cv*-TA Y322 (highlighted).

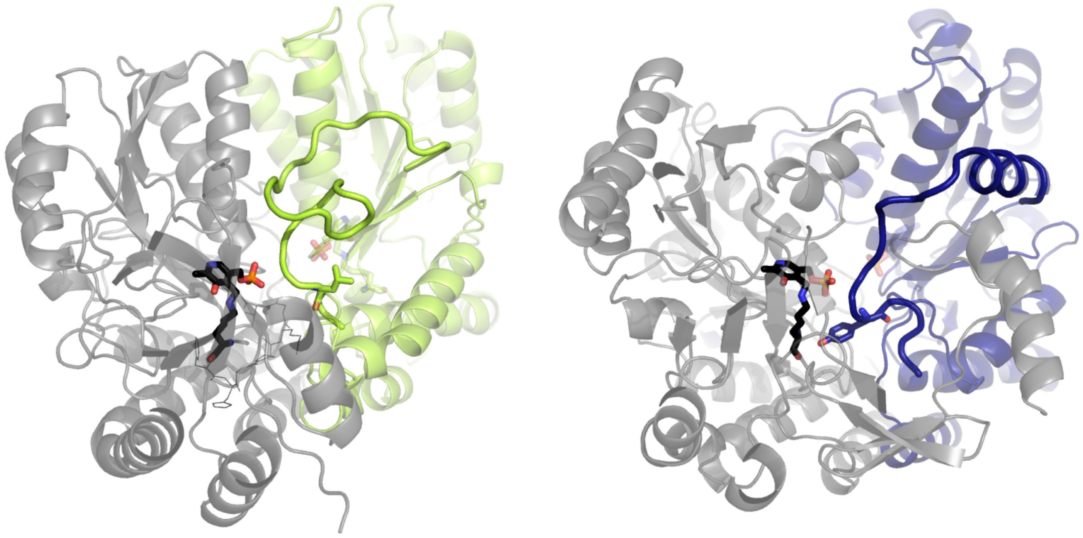

Holo-*Ht*-DS　　　　Holo-*Cv*-TA

Figure S14. Comparison of the monomeric architecture of the Holo-form of the *Ht*-DS (on the left, PDB ID: 5ZSP) and *Cv*-TA (on the right, PDB ID: 4A6T) as viewed from the dimer interface. The Lys-PLP moiety is represented as black sticks, while the *Cv*-TA Y322 and its closest spatial correspondent in *Ht*-DS are represented as sticks colored in blue or green, respectively. The interfacial loops are represented as tubes, while the regions preceding and following this structure segments are represented as colored ribbons.

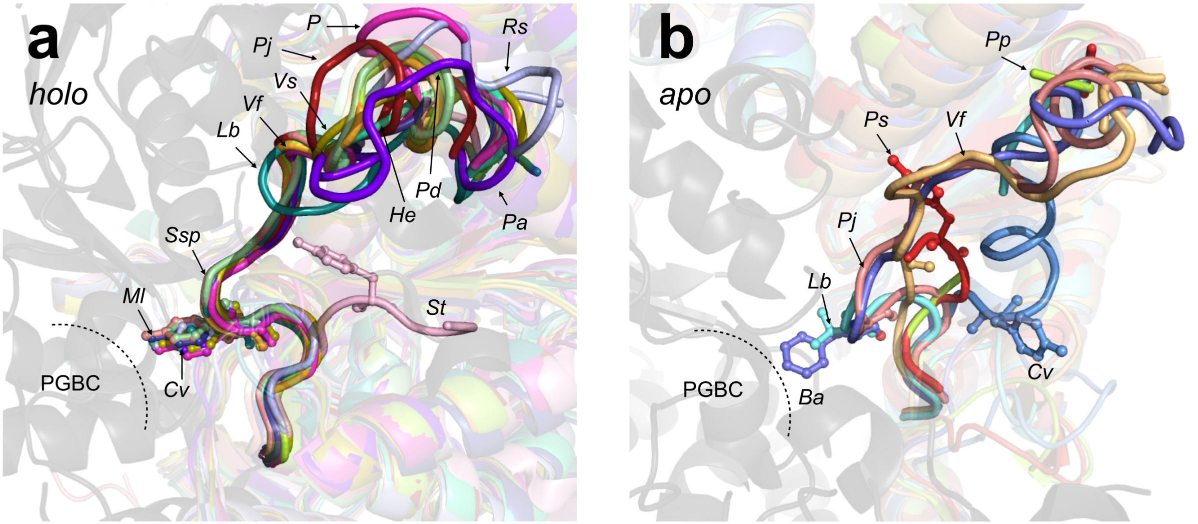

Figure S15. Multiple structural alignments of the holo- (panel a) and apo- (panel b) crystal structures considered in the working set. The interfacial loop of each structure is shown as solid tube, while the structural analogue to the *Cv*-TA is represented as ball-and-stick. The surface of the PGBCs is virtually represented with a dotted black line. Individual structures are identified by explicit labels reporting the enzyme abbreviations introduced in Table S4. All PDB codes are reported in Table S4.

References

1. Humble, M. S. *et al.* Crystal structures of the Chromobacterium violaceumω-transaminase reveal major structural rearrangements upon binding of coenzyme PLP: Structural states of C. violaceumω-transaminase. *FEBS J.* **279**, 779–792 (2012).22.

2. Sayer, C., Isupov, M. N., Westlake, A. & Littlechild, J. A. Structural studies of *Pseudomonas* and *Chromobacterium* ω-aminotransferases provide insights into their differing substrate specificity. *Acta Crystallogr. D Biol. Crystallogr.* **69**, 564–576 (2013).
